# Transcriptome profiling and network enrichment analyses identify subtype-specific therapeutic gene targets for breast cancer and their microRNA regulatory networks

**DOI:** 10.1038/s41419-023-05908-8

**Published:** 2023-07-12

**Authors:** Ramesh Elango, Sameera Rashid, Radhakrishnan Vishnubalaji, Reem Al-Sarraf, Mohammed Akhtar, Khalid Ouararhni, Julie Decock, Omar M. E. Albagha, Nehad M. Alajez

**Affiliations:** 1 Translational Cancer and Immunity Center (TCIC), Qatar Biomedical Research Institute (QBRI), Hamad Bin Khalifa University (HBKU), Qatar Foundation (QF), Doha, Qatar; 2 Department of Laboratory Medicine and Pathology (DLMP), Hamad Medical Corporation (HMC), Doha, Qatar; 3 The Christie NHS Foundation Trust, Manchester, UK; 4 Genomics Core Facility, Qatar Biomedical Research Institute (QBRI), Hamad Bin Khalifa University, Qatar Foundation, Doha, Qatar; 5 College of Health & Life Sciences, Hamad Bin Khalifa University (HBKU), Qatar Foundation (QF), Doha, Qatar; 6 Centre for Genomics and Experimental Medicine, Institute of Genetics and Cancer, University of Edinburgh, Edinburgh, UK

**Keywords:** Breast cancer, Breast cancer

## Abstract

Previous studies have suggested that breast cancer (BC) from the Middle East and North Africa (MENA) is presented at younger age with advanced tumor stage, indicating underlying biological differences. Given the scant transcriptomic data on BC from the MENA region and to better understand the biology of this disease, we performed mRNA and microRNA (miRNA) transcriptomic profiling on a local cohort of BC (*n* = 96) from Qatar. Our data revealed the differentially expressed genes and miRNAs as function of BC molecular subtypes (HR^+^, HER2^+^, HER2^+^HR^+^, and TNBC), tumor grade (GIII vs GI-II), patients’ age (young (≤40) vs old (>40)), and ethnicity (MENA vs non-MENA). Our profiling data revealed close similarity between TNBC and HER2^+^, while the transcriptome of HER2^+^HR^+^ tumor was resemblant of that from HR^+^ tumors. Network analysis identified complex miRNA-mRNA regulatory networks in each BC molecular subtype, in high *vs* low grade tumors, in tumors from young vs old patients, and in tumors from MENA vs non-MENA, thus implicating miRNA-mediated gene regulation as an essential mechanism in shaping the transcriptome of BC. Integration of our transcriptomic data with CRISPR-Cas9 functional screen data and the OncoKB database identified numerous dependencies and therapeutic vulnerabilities in each BC molecular subtype, while CDC123 was functionally validated as potential therapeutic target for TNBC. Cox regression survival analyses identified mRNA and miRNA-based signatures predicative of worse and better relapse free survival (RFS), which were validated in larger BC cohorts. Our data provides comprehensive transcriptomic profiling and unraveled the miRNA-mRNA regulatory networks in BC patients from the region and identified novel actionable gene targets, employing integrated approach. Findings from the current study have potential implications to improve the current standard-of-care for BC from the MENA as well as patients from other ethnicities.

## Introduction

Breast cancer (BC) is the most prevalent malignant disease and the second foremost cause of death among women worldwide [1]. Clinical management of BC is dependent on tumor size, nodal status, distant metastasis, and BC molecular subtypes. BC are clinically stratified based on estrogen receptor (ER), progesterone receptor (PR), and HER2 expression into three major subtypes namely: Luminal (ER^+^, PR^+/−^), HER2^+^ (HER2^+^, ER^+/−^, PR^+/−^) and triple negative (TNBC; ER^−^/PR^−^/HER2^−^) correlating with prognosis where the expression of those biomarkers plays essential role in defining treatment strategies [2]. Additionally, BC can be classified according to PAM50 gene signature into five intrinsic molecular subtypes: Luminal A (LumA; ER^+^/PR^+^/HER2^–^/ki67 > 14%), Luminal B (LumB; ER^+^/PR^–^/^+^HER2^–^/ki67 > 14%), HER2-enriched, basal-like, and normal-like, with each molecular subtype exhibiting distinct features and prognosis [3, 4]. GLOBOCAN 2020 highlighted an estimated 2.3 million new cases and 685,000 deaths from BC worldwide [5]. The age standardized incidence rates (ASRs) for BC are on the rise in several countries, including the MENA region, where the annual reported ASR rates ranged between 9.5 and 50 cases per 100,000 women [6]. In Qatar, the ASR for BC was 42.7 per 100,000, slightly lower than the world’s ASRs (47.8 per 100,000) [5]. Najjar et al. reported BC patients from the MENA to be diagnosed at younger age and with more advanced tumor stage at first presentation [7]. Comparing BC from Northern and Southern Mediterranean regions revealed tumors from the south to be predominantly more aggressive luminal B, compared to luminal A from the North [8]. This data underscores the need for better understanding of the biology of BC from the MENA region for improved clinical care.

Currently, there are only few studies focusing on transcriptomic profiling of BC from the region, which are limited in cohort size and aimed at addressing specific questions [9, 10]. A recent population-based study by Saad et al. revealed significant difference in cancer predisposition genes and polygenic risk scores across different ancestries from Qatar [11]. In the current study, we performed comprehensive mRNA and miRNA transcriptomic profiling on 96 BC tumor tissue samples from Qatar and identified the miRNA-mRNA regulatory networks as function of BC molecular subtype, tumor grade, age, and ethnicity. Additionally, survival analysis on the same cohort identified gene- and miRNA-based signatures predictive of relapse free survival (RFS). Employing CRISPR-Cas9 functional screen data, we identified a number of promising therapeutic targets for each BC molecular subtype. According to the OncoKB database, specific drugs targeting several of our identified gene targets are already in clinical testing for various cancer types. However, for several of the identified targets, there are currently no therapeutic approaches available, hence the development of small molecule inhibitors targeting those candidate genes is needed. Our data provide a framework for future development and testing of novel prognostic biomarkers and therapeutic interventions for BC from the region, which could also be beneficial to BC patients from other ethnicities.

## Materials and methods

### Ethics approval and consent to participate

This study was performed under ethical approval (MRC-01–19–142) from Hamad Medical Corporation (HMC) and ethical approval (QBRI-IRB 2020–09–035) from Qatar Biomedical Research Institute (QBRI). Written informed consent was not required since the study was conducted on archived FFPE surgical specimens.

### Patient characteristics

Archived Formalin-Fixed Paraffin-Embedded (FFPE) tissue from 96-BC patients was obtained from the Department of Laboratory Medicine and Pathology (DLMP), HMC, Doha, Qatar. Clinical and survival data were retrieved from electronic medical records from HMC. The inclusion criteria were female patients with invasive breast carcinoma who underwent surgical excision of the primary tumor at HMC, Qatar between 2008 and 2013. The exclusion criteria were post-neoadjuvant chemotherapy status, in-situ malignancy, male gender, recurrent BC, sarcoma and metastatic carcinoma from non-breast primary tumor. Patients’ and tumor characteristics are listed in Table 1.

**Table 1:** Clinical characteristics of study cohort.

Ethnicity	
MENA	60
Non-MENA	36
Age (median)	23–72 (41)
BC molecular subtype	
HR^+^	35 (36%)
HER2^+^	15 (16%)
HER2^+^HR^+^	31 (32%)
TNBC	15 (16%)
SBR grade	
I	11 (11%)
II	37 (39%)
III	48 (50%)

### Total RNA isolation from FFPE tissues

Total RNA was extracted from FFPE core punches using the recover all total nucleic acid isolation kit (Ambion Inc., Life Technologies, USA) according to the manufacturer’s protocol with slight modifications as we previously described [12]. Briefly, core punches (<35 mg) were transferred into a mortar containing liquid nitrogen and were grinded thoroughly using a pestle, followed by deparaffinization through incubation in xylene for 3 min at 50 °C, centrifugation, and two washes in 100% ethanol. The resulting pellets were then vacuum-dried. Proteins were degraded using protease enzyme, followed by 15 min incubation at 50 °C followed by another 15 min incubation at 80 °C. Nucleic acids were then captured using column, followed by DNAse treatment, and RNA was finally eluted in nuclease-free water. The concentration and purity of extracted RNA was measured using NanoDrop 2000 (Thermo Scientific, DE, USA) and RNA was stored at −80 °C until further use.

### Quality assessment of RNA

The quality and quantity of extracted RNA was measured using on-chip electrophoresis utilizing the Agilent RNA 6000 Nano Kit (Agilent Technologies, CA, USA) and Agilent 2100 Bioanalyzer (Agilent Technologies) as per the manufacturer’s instructions.

### Total RNA library preparation and RNA sequencing

Total RNA was used for library preparation using TruSeq Stranded Total RNA Library (Cat no. 20020597, Illumina Inc., San Diego, CA, USA) following the manufacturer’s protocol. Briefly, 500 ng of total RNA was subjected to rRNA depletion, without fragmentation. The first-strand cDNA synthesis was performed using random hexamers and SuperScript II Reverse Transcriptase (Cat#: 18064014) from Thermo Fisher Scientific (Thermo Fisher Scientific, Waltham, MA). The second cDNA strand synthesis was performed by substitution of dTTP with dUTP. The double-stranded cDNA was end-repaired and adenylated. Barcoded DNA adapters were ligated to both ends of the double-stranded cDNA and then amplified. The library’s quality was checked using an Agilent 2100 Bioanalyzer system and quantified using Qubit system. The libraries were pooled, clustered on a cBot platform, and sequenced on an Illumina HiSeq 4000 at approximately 50 million paired end reads (2 × 75 bp) per sample. Total RNA transcptomic data generated in this study have been deposited in the SRA repository under BioProject number PRJNA953015.

### Total RNA‑Seq data and bioinformatics analysis

Pair-end FASTQ files were subsequently aligned to the GRCh38 reference genome using built-in module and default settings in CLC genomics workbench v21.0.5. Expression data (total counts) were then imported into iDEP.951 and were first normalized (CPM, count per million) and then data transformation was conducted using EdgeR (log2(CPM+c)) as described before [13]. mRNAs with minimum expression of 5 CPM (count per million) in at least 10 samples were retained. Hierarchical clustering was conducted using correlation distance and average linkage. DESeq2 was used to identify Differential Expressed Genes (DEGs). PAM50 classification was performed using classifieRb as described by Quinn et al. [14]. Differential expression analysis based on PAM50 classification was conducted using iDEP.951 as described above.

### miRNA library preparation and bioinformatics analysis

For small RNA library preparation, 100 ng of RNA was subjected to 3′ ligation followed by 5′ ligation and reverse transcription as we described before [15]. Subsequently, QIAseq miRNA NGS (QMN) bead preparation, cDNA cleanup, and finally library amplification was carried out using tube indices (QIAseq miRNA NGS 96 Index IL kit, QIAGEN, Hilden, Germany). The yield of cDNA libraries was quantified using Qubit dsDNA HS assay kit (Invitrogen) and size distribution of the cDNA libraries were determined using the Agilent 2100 Bioanalyzer DNA1000 chip (Agilent Technologies). Libraries were subsequently pooled and were subjected to sequencing using Illumina platform as described above. miRNA transcriptomic data generated in this study have been deposited in the SRA repository under BioProject number PRJNA954402.

For miRNA analysis, FASTQ files were mapped to the miRBase v22 database and miRNA expression (total counts) was estimated using built-in small RNA analysis workflow and default settings in CLC genomics workbench 20.2. Data were then imported into iDEP.951 and were first normalized (CPM, count per million) and then log transformed using EdgeR (log2(CPM+c)). miRNAs with minimum expression of 5 CPM (count per million) in at least 10 samples were retained. Hierarchical clustering and identification of differentially expressed miRNAs (DEMs) was conducted as described above. To identify bone fide gene targets for differentially expressed miRNAs, the microRNA Target Filter in IPA was employed as we previously described [15] by crossing the list of differentially expressed miRNAs and mRNAs and integration with CLC miRNA target database comprised of in silico predicted in addition to targets those were experimentally validated.

### Gene enrichment and functional annotation analysis

DEGs based on DESeq2 analysis were then subjected to enrichment analysis using gene ontology (GO) biological processes. Enrichment heatmaps and GO enrichment scores were plotted using GraphPad prims v9.0.

### MarkerFinder analysis

To identify unique gene markers associated with each of the main BC molecular subtype (HR^+^, HER2^+^, and TNBC), the MarkerFinder algorithm was employed [16]. DEGs in HR^+^ vs TNBC, HR^+^ vs HER2^+^, HER2^+^ vs TNBC were used as input to identify genes unique to each BC molecular subtype, employing correlation analysis.

### Discriminant analyses

To assess the ability of predicted variables (genes identified from the MarkerFinder analysis) to distinguish between HR^+^, HER2^+^, and TNBC, discriminant analyses were performed using an OPLS-DA classifier using Soft Independent Modeling by Class Analogy (SIMCA) software (version 16; Umetrics, Sweden) as we described before [17]. The performance of the model was assessed by generating the receiver operating characteristic (ROC) curve and determining the area under the curve (AUC) value. The sensitivity and specificity constants of the test were determined based on similar classification scores by OPLS-DA. The model was then validated in another BC cohort from the cancer genome atlas (TCGA) database.

### Quantitative real time polymerase chain reaction (qPCR) gene validation

Selected gene candidates based on DE analysis were then subjected to qRT-PCR to validate their expression in samples from the same cohort. Briefly, 500 ng of RNA was reverse transcribed to cDNA using the high-capacity cDNA Reverse Transcription kit (Applied Biosystems, Foster City, CA, USA). qRT-PCR was done using primer pairs listed in Table S1, and PowerUp™ SYBR™ Green Master Mix (Applied Biosystems) on QuantStudio 7/6 Flex qPCR (Applied Biosystems). Relative levels of transcripts were determined from their respective CT values normalized against GAPDH transcript levels and were presented as –(delta CT).

### Validation in additional BC datasets

Validation of the prognostic value of mRNA-based gene signatures was conducted on 2023 BC samples from the KMplot database [18]. Correlation analysis between miRNA and mRNA expression were conducted using the StarBase V3.0 database (https://starbase.sysu.edu.cn/panMirCoExp.php) [19]. To provide an insight on potential role of CDC123 in TNBC, differential expression and gene set enrichment analysis was performed on cohort of 360 TNBC retrieved from the sequence read archive (SRA) database (https://www.ncbi.nlm.nih.gov/sra/SRP157974). Paired-end RNA-seq FASTQ files were subsequently pseudo aligned to the GENCODE release (v33) using KALLISTO 0.4.2.1 as described before [20]. The cohort was divided into high vs low based on median CDC123 expression and were then subjected to differential expression (1.5 fc, *p* < 0.05 FDR) and enrichment analysis in iDEP.951 as described above.

### TNBC cell culture and siRNA transfection

Human TNBC cell lines (BT-549 and MDA-MB-231) were maintained in DMEM (Dulbecco’s modified Eagle’s medium) supplemented with D-glucose 4500 mg/L, 2–4 mM L-glutamine, 10% fetal bovine serum, and 1× penicillin-streptomycin (Pen-Strep). All reagents were purchased from GIBCO-Invitrogen, Waltham, MA, USA). Cells were grown at 37 °C in humidified CO2 (5%) incubator. The scrambled siRNA control and ON-TARGETplus SMARTpool siRNA targeting human CDC123 were purchased from Dharmacon (Lafayette, CO, USA). Transfection was performed using a reverse transfection approach. Briefly, siRNA at 30 nM final concentration was diluted in 50 μL of Opti-MEM (11058-021; GIBCO, Carlsbad, CA, USA), and 1 μl of Lipofectamine 2000 (cat. no. 52758; Invitrogen) was diluted in 50 μl of OPTI-MEM. The diluted siRNA and Lipofectamine 2000 were mixed and were incubated at room temperature for 20 min. Twenty microliters of transfection mixture was added to the tissue culture plate, and subsequently 10,000 cells in 60 μl transfection medium (complete DMEM without Pen-Strep) were added to each well. After 24 h, the transfection cocktail was replaced with complete DMEM. To validate gene knockdown efficiency, total RNA was isolated from siCtrl and siCDC123 transfected MDA-MB-231 and BT-549 TNBC cells at 48 h using miRNeasy Mini kit (cat. no: 217004, Qiagen, Germany). The concentration and purity of extracted RNA was measured using NanoDrop 2000 (Thermo Scientific, DE, USA). Subsequently, 1,000 ng of total RNA was reverse transcribed using high-capacity cDNA reverse transcript kit (Applied Biosystems, Foster City, CA, USA) followed by qRT-PCR as described above.

### Colony formation unity (CFU) and Organoid 3-dimensional (3D) culture

Transfected cells were cultured for 48 h and were then maintained alone or in combination with paclitaxel (10 nM) or doxorubicin (10 nM). On day 7, cells were fixed with 4% PFA for 5 min followed by washing twice in PBS and stained with crystal violet (0.1% in 10% EtOH) for 10 min at room temperature. The images were taken and compared with appropriate controls. Subsequently, plates were air-dried at room temperature, and CFUs were quantified by dissolving crystal violet in 5% SDS and measuring absorbance at 590 nm. The experiments were repeated twice, and data are represented as mean ± SD from four technical replicas.

The 3D organoid cultures were conducted as we previously described [21]. Briefly, 250,000 transfected cells were mixed with overnight thawed Matrigel (Corning cat. no. 356231; Growth Factor Reduced (GFR) Basement Membrane Matrix). Subsequently, multiple drops of cell suspension were plated in pre-warmed (37 °C) 60 mm Ultra-Low Attachment Culture Dish (Corning; 3261). Dishes were then placed upside-down in a 37 °C, 5% CO_2_ cell culture incubator to allow the droplets to solidify for 20 min, before adding 4–5 mL of expansion medium alone or supplemented with paclitaxel (10 nM) or doxorubicin (10 nM). Seven days later, organoid formation was observed under the microscope.

### Survival analysis

To identify genes associated with patients’ RFS, the normalized gene expression data (log2) were subjected to cox regression survival analysis as we previously described [16]. Genes significantly associated with RFS were initially identified using univariate Cox regression analysis in IBM SPSS statistics v26. Significant genes were then subjected to multivariate Cox regression survival analysis, to adjust for potential confounding factors (molecular subtype, tumor grade, and age). The Benjamini-Hochberg false discovery rate (FDR) multiple testing method was used to correct for type I error (https://tools.carbocation.com/FDR). This analysis identified genes associated with worse or better prognosis. The identified genes were subsequently subjected to a forward stepwise Cox regression analysis in which variables with stepwise probability <0.05 were included in the model, while variables with probability of >0.1 were excluded to identify gene signatures predicative of better and worse RFS. The equation for worse survival mRNA signature was Score = (1.3*ENSG00000109458(exp) + 1.4*ENSG00000109519(exp) + 2.9*ENSG00000131100(exp) + 0.67*ENSG00000145649(exp) + 0.4*ENSG00000177614(exp) + 0.57*ENSG00000187398(exp) + 0.52*ENSG00000205592(exp). Curve comparison was conducted by dividing the cohort into high and low, according to median score. The equation for a better survival associated mRNA signature was Score = (4.3*ENSG00000107651(exp) + 1.6*ENSG00000119950(exp) + 2.4*ENSG00000134369(exp) + 0.56*ENSG00000141750(exp) + 1.3*ENSG00000184117(exp) + 1.6* ENSG00000188352(exp) + 1.0* ENSG00000213967(exp) + 1.2* ENSG00000254413(exp). The equation for better survival based on miRNA expression was Score = (0.7*hsa-miR-5683(exp) + 0.9*hsa-miR-491-5p(exp) + 0.6*sa-miR-181C-5p(exp), while calculating the score for worse survival based on miRNA expression was conducted as following; Score = (1.9*hsa-miR-887-3p(exp) + 2.4*hsa-miR-708-3p(exp) + 0.01*hsa-miR-671-5p(exp) + 0.6*hsa-miR-146a-3p(exp).

### Statistical analysis

Statistical analyses for DEGs and DEMs were conducted in iDEP.951. Fold change (2.0) and FDR adjusted *p* value < 0.05 was used as cutoff, unless stated otherwise. Survival analyses were conducted in IBM SPSS v26.0 and log-rank *p* value of < 0.05 was considered significant. Graphing and pairwise statistical analyses were conducted in GraphPad prism v9.

## Results

### Transcriptomic profiling of BC as function of molecular subtypes, tumor grade, age, and ethnicity

**Fig. 1:**
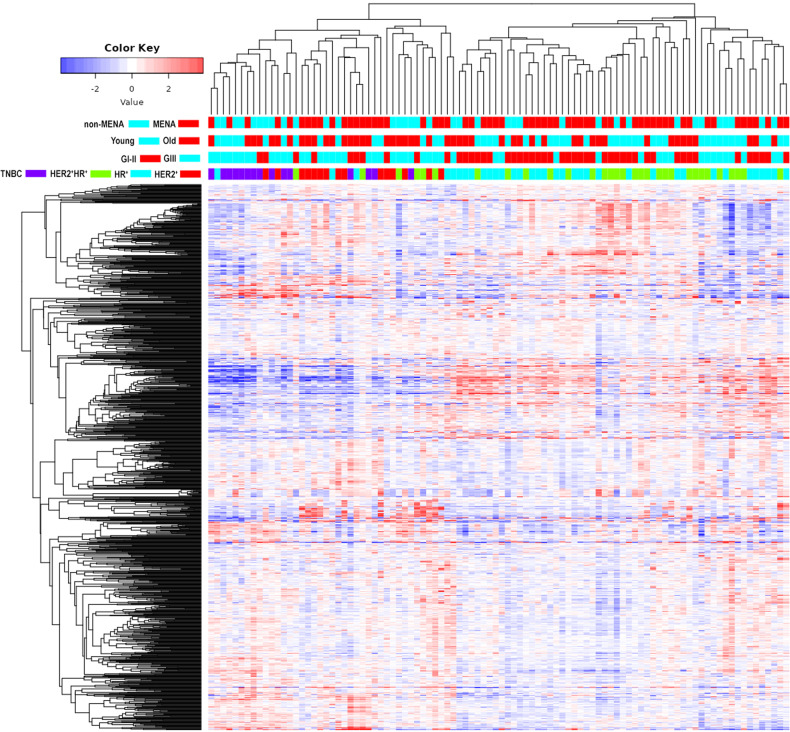
Hierarchical clustering of BC based on mRNA expression. Heatmap depicting clustering of 96 BC patients as function of BC molecular subtype (HR^+^, HER2^+^, HER2^+^HR^+^, and TNBC), tumor grade (GIII vs GI–II), age (young (≤40), old (>40)), and ethnicity (MENA = 60, non-MENA = 36) based on top 1000 most variable genes. Color scale depicts the expression level of each gene. Each row represents an mRNA, and each column represents a sample.

**Fig. 2:**
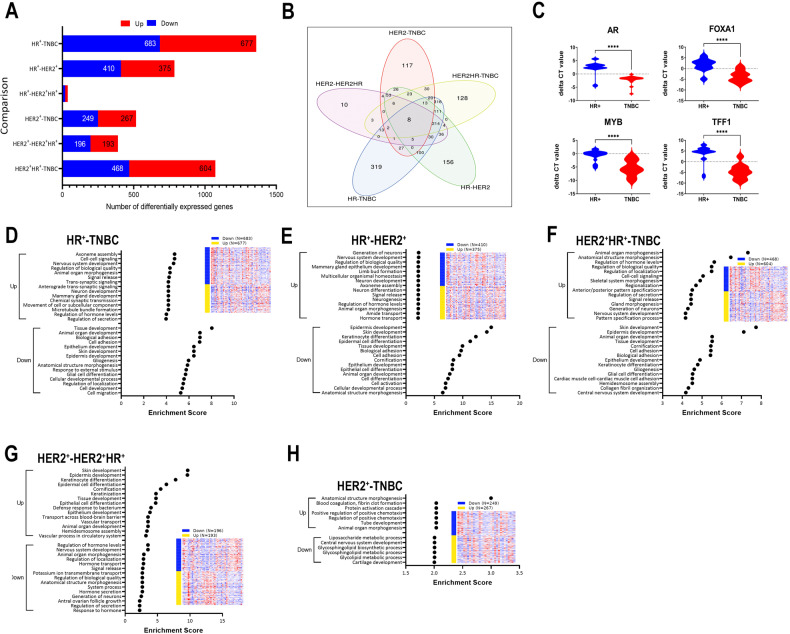
Differential gene expression analysis as function of BC molecular subtypes. **A** Bar chart depicting the number of DEGs in HR^+^ vs TNBC, HR^+^ vs HER2^+^HR^+^, HR^+^ vs HER2^+^, HER2^+^HR^+^ vs TNBC, HER2^+^ vs TNBC, and in HER2^+^ vs HER2^+^HR^+^ after adjusting for age (young (≤40) vs old (>40)), tumor grade (GIII vs GI-II), and ethnicity (MENA vs non-MENA) using 2.0 fold change (fc) and adjusted FDR (*p* < 0.05). **B** Venn diagram depicting the overlap between DEGs for the indicated comparisons. **C** Validation of selected upregulated gene (AR, FOXA1, MYB, and TFF1) in HR^+^ (*n* = 20) vs TNBC (*n* = 20) using qRT-PCR where each sample was run in duplicate. Data are presented as violin plot. ****p < 0.0001. **D**–**H** Gene ontology (GO) biological processes functional enrichment analysis of upregulated and downregulated genes for each comparison. X-axis depicts enrichment score, while y-axis represents the enriched GO functional category.

To provide a thorough characterization of the molecular alterations in BC as function of molecular subtypes (HR^+^, HER2^+^, HER2^+^HR^+^, and TNBC), tumor grade (GIII vs GI–II), patients age at presentation (young (≤40) vs old (>40)), and ethnicity (MENA vs non-MENA), a total of 96 well-characterized BC samples were subjected to total RNA profiling. Hierarchical clustering based on top 1000 variable mRNAs revealed strongest association with BC molecular subtypes, where TNBC and HER2^+^ clustered separately from HR^+^ and HER2^+^HR^+^ tumors (Fig. 1). In contrast, tumor grade, age and ethnicity had minimal impact on clustering. We subsequently sought to identify the differentially expressed genes (DEGs) in each molecular subtype (HR^+^ vs TNBC, HR^+^ vs HER2^+^, HR^+^ vs HER2^+^HR^+^, HER2^+^ vs TNBC, HER2^+^ vs HER2^+^HR^+^, and HER2^+^HR^+^ vs TNBC) after adjusting for age (young vs old), tumor grade (GIII vs GI-II), and ethnicity (MENA vs non-MENA) using 2.0 fold change (fc) and adjusted false discovery rate (FDR) of *p* < 0.05. DEG analysis identified 677 upregulated and 683 downregulated genes in HR^+^ vs TNBC, while 375 genes were upregulated and 410 genes were downregulated in HR^+^ vs HER2^+^ (Fig. 2A, and Table S2). When comparing HER2^+^ vs TNBC, 267 genes were upregulated, while 249 genes were downregulated (Fig. 2A and Table S2). Comparing HER2^+^ and HER2^+^HR^+^, we identified 193 upregulated and 196 downregulated genes (Fig. 2A and Table S2). A large number of differentially expressed genes was observed when comparing HER2^+^HR^+^ vs TNBC (604 upregulated and 468 downregulated), while the least number of differentially expressed genes was observed when comparing HR^+^ vs HER2^+^HR^+^ (18 upregulated and 19 downregulated) as illustrated in Fig. 2A and Table S2. Venn analysis revealed that largest commonality in DEGs was seen when comparing HR^+^ vs TNBC and HER2^+^HR^+^ vs TNBC, which is most likely due to a large similarity between HR^+^ and HER2^+^HR^+^ BC (Fig. 2B), which is in line with the observation that only a few genes were differentially expressed when comparing HR^+^ vs HER2^+^HR^+^ (Fig. 2A and Table S2). These observations suggest that molecular changes in gene expression in HER2^+^HR^+^ may be mostly driven by HR status. The expression of four representative genes (androgen receptor (AR), Forkhead Box A1 (FOXA1), MYB Proto-Oncogene, Transcription Factor (MYB), Trefoil Factor 1 (TFF1) identified from differential expression analysis with known expression in ER^+^ BC, was validated in HR^+^ vs TNBC tumors using qRT-PCR (Fig. 2C). When comparing HR^+^ vs TNBC, gene ontology (GO) analysis revealed enrichment of numerous functional categories in HR^+^ tumors, including axoneme assembly, nervous system development, mammary gland development, regulation of hormone and regulation of secretion, while categories associated with tissue development, cell adhesion, epithelial and skin development, as well as cell movement were more enriched in TNBC (Fig. 2D). Similar enrichment patterns were seen when comparing HR^+^ vs HER2^+^, while functional categories enriched in HER2^+^ tumors were associated with epidermis, skin, and epithelial development, as well as cell adhesion in addition to numerous other categories (Fig. 2E). Functional categories enriched in HER2^+^HR^+^ compared to TNBC (Fig. 2F) were similar, to some extent, to those enriched in HR^+^ BC as compared to TNBC (Fig. 2D). Similar patterns of enrichment were seen when comparing HER2^+^ vs HER2^+^HR^+^ (Fig. 2G) to those seen when comparing HER2^+^ vs HR^+^ (Fig. 2E), again suggesting the molecular profiles of HER2^+^HR^+^ to be very similar to the HR^+^ tumors. Minimal enrichment was seen when comparing HER2^+^ vs TNBC, which is concordant with the number of DEGs between the two BC molecule subtypes (Fig. 2H). We subsequently employed the PAM50 classification and stratified the cohort into LumA, LumB, HER2, basal-like, and normal-like using the ClassifieRb algorithm as described before [14]. We observed the basal-like and HER2 tumors to cluster separate from LumA, LumB, and normal-like (Fig. S1A). Differential expression analysis based on PAM50 classification revealed the differentially expressed mRNAs in each intrinsic subtype, while adjusting for age (young vs old), tumor grade (GIII vs GI-II), and ethnicity (MENA vs non-MENA) using 2.0-fold change (fc) and adjusted FDR of p < 0.05 (Fig. S1B and Table S3). Differential expression analysis based on PAM50 classification identified a larger number of differentially expressed mRNAs compared to classification based on hormone receptor and HER2 status, while largest difference was seen when comparing LumA and basal BC.

### Identification of unique gene markers enriched in each BC molecular subtype

**Fig. 3:**
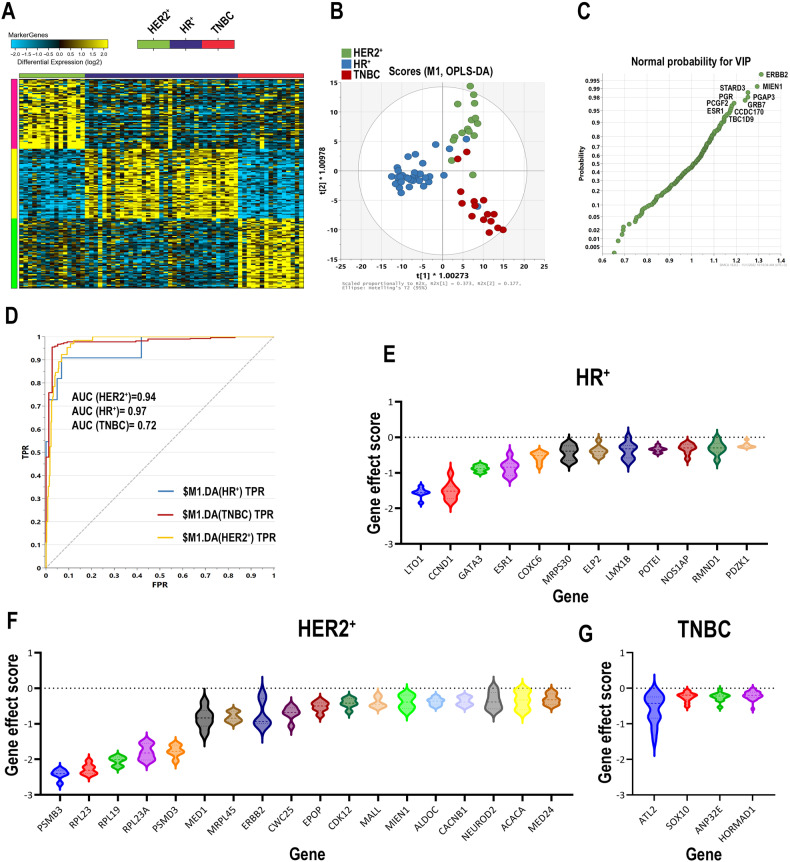
Identification of genes most associated with main BC molecular subtypes. **A** Heatmap depicting the enriched gene markers associated with HER2^+^ (*n* = 15), HR^+^ (*n* = 20), and TNBC (*n* = 15) BC molecular subtypes employing the MarkerFinder algorithm. Only genes exhibiting >2.0 fc and adjusted FDR < 0.05 in each molecular subtypes based on DEG analysis were used as input **B** Orthogonal partial least squares-discriminant analysis (OPLS-DA) employing the expression of top 60 identified gene markers for each molecular subtype from **A** using SIMCA. **C** Variable influence on projection (VIP) plot depicting the contribution of each marker in the model. The top 10 genes with highest influence are shown. **D** Validation of the discriminative power of the identified gene markers in another BC cohort (HR^+^ (*n* = 269), HER2^+^ (*n* = 11^)^, and TNBC (*n* = 65)) form TCGA BRCA using ROC analysis in SIMCA. Violin plots illustrating the perturbational gene effects of twelve HR^+^ essential genes in eight HR^+^ BC cell lines **E**, eighteen HER2^+^ essential genes in six HER2^+^ BC cell lines **F**, and four TNBC essential genes in twenty-three TNBC cell lines **G**. CRISPR-Cas9 perturbational gene effects data were retrieved from the Dependency Map database. Y-axis represent the perturbational effect scores for the indicated genes.

The data presented thus far highlighted the differentially expressed genes between various BC molecular subtypes, however our DEG analysis revealed large similarities when comparing for instance HR^+^ vs TNBC and HR^+^ vs HER2^+^. Therefore, the identification of unique gene markers and their expression pattern in each main BC molecular subtype is empirical for better disease stratification and identification of novel therapeutic targets. In this context, we first identified the gene list that were most associated with each BC molecule subtype based on differential expression analysis by identifying the genes that were upregulated in HR^+^ vs TNBC and in HR^+^ vs HER2^+^ (2.0 fc, adjusted FDR < 0.05), and marked those as HR-associated genes (Fig. S2). Similarly, we then identified the genes that were most associated with HER2^+^ (upregulated in HER2^+^ vs HR^+^ and in HER2^+^ vs TNBC) as well as those that were most associated with TNBC (upregulated in TNBC vs HR^+^ and in TNBC vs HER2^+^), figure S2. Using this strategy, we identified 284 genes enriched in HR^+^, 92 genes enriched in HER2^+^, and 158 genes enriched in TNBC. This complied gene list containing 534 genes was then subjected to the MarkerFinder algorithm to identify the top genes associated with each BC molecular subtype, as we described before [22]. The heatmap presentation of enriched genes in each of the three BC molecule subtypes is shown in Fig. 3A. The list of enriched genes in each molecular subtype is provided in Table S4. To assess the ability of the identified gene markers to discriminate different BC molecular subtypes, we employed OPLS-DA, which revealed remarkable segregation of the HR^+^, HER2^+^, and TNBC based on the identified gene markers (Fig. 3B). Using Variable influence on projection (VIP) analysis, we identified the contribution of each variable in the model with the top ten predictors illustrated in Fig. 3C and are listed in Table S5. ROC analysis revealed excellent performance of the identified 180 gene classifiers in discriminating the three BC molecular subtypes (AUC (HER2^+^) = 0.94, AUC (HR^+^) = 0.97, and AUC (TNBC) = 0.97) in an independent validation cohort from BRCA TCGA dataset (HR^+^ (*n* = 269), HER2^+^ (*n* = 11), and TNBC (*n* = 65)) as shown in Fig. 3D.

**Fig. 4:**
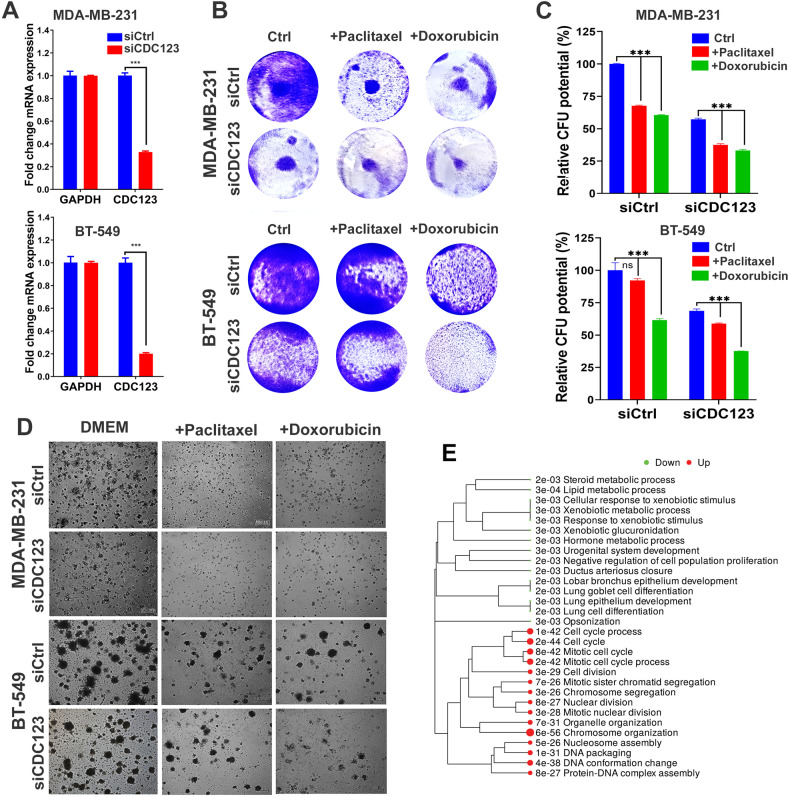
Targeted depletion of CDC123 inhibits TNBC colony and 3D organoid formation. **A** qRT-PCR demonstrating efficient CDC123 know down in MDA-MB-231 (upper panel) and BT-549 (lower panel) TNBC models. Data are presented as mean ± SE, *n* = 3. **B** Suppression of CFU potential of MDA-MB-231 (upper panel) and BT-549 (lower panel) in response to CDC123 depletion as single agent or in combination with paclitaxel (10 nM) or Doxorubicin (10 nM) on day 7. **C** Quantitative analysis of relative CFU potential under each experimental condition. Data are presented as mean ± SD, *n* = 4. **D** Inhibition of 3D organoid formation of MDA-MB-231 (upper panels) and BT-549 (lower panels) TNBC models in response to CDC123 depletion; scale bar = 100 µM. **E** GO enrichment tree in a cohort of 360 TNBC patients divided into high vs low according to median CDC123 expression. Enrichment *p* value is indicated for each functional category. ***p < 0.0005.

We subsequently sought to identify potential therapeutic targets for each of the three BC molecular subtypes. The differentially expressed genes from each BC molecular subtype (Fig. S2) were crossed with CRISPR-Cas9 perturbational gene effects data from the Achilles project [23]. Using this approach, we identified twelve HR^+^ (LTO1, CCND1, GATA3, ESR1, COXC6, MRPS30, ELP2, LMX1B, POTEI, NOS1AP, RMND1, and PDZK1; Fig. 3E), eighteen HER2^+^ (PSMB3, RPL23, RPL19, RPL23A, PSMD3, MED1, MRPL45, ERBB2, CWC25, EPOP, CDK12, MALL, MIEN1, ALDOC, CACNB1, NEUROD2, ACACA, and MED24; Fig. 3F), and four TNBC (ATL2, SOX10, ANP32E, and HORMAD1; Fig. 3G) essential genes. The limited number of TNBC essential genes could be attributed to the large similarity between TNBC and HER2^+^ (i.e., only few genes were unique to TNBC), alternatively when comparing the enriched genes in TNBC vs HR^+^ BC only, we identified twenty TNBC essential genes (CDC123, CENPW, PWP2, ATP1A1, NOP2, YBX1, RPP4, PPP1CB, TSPYL5, HMGA1, SLC7A5, ATL2, CAD, CPAMD8, FA.BP5, SDC1, PPP1R1A, SLC2A1, MRPS17, and SOX9), Fig. S3. Targeted depletion of CDC123 inhibited CFU potential of MDA-MB-231 and BT-549 TNBC models as single agent or in combination with paclitaxel or doxorubicin, thus corroborating the CRISPR-Cas9 screen data (Fig. 4A, B). Quantitative analysis revealed significant inhibition of CFU potential of MDA-MB-231 and BT-549 TNBC cell models in response to CDC123 depletion (Fig. 4C). Similarly, CDC123 loss inhibited the organotypic growth of MDA-MB-231 and BT-549 TNBC cell models under 3D culture condition, which was further enhanced when combined with paclitaxel or doxorubicin (Fig. 4D). To provide perspective on potential role of CDC123 in TNBC tumors, differential expression (1.5 fc, *p* < 0.05 FDR) in CDC123^high^ (*n* = 180) vs CDC123^low^ (*n* = 180) TNBC patients and gene set enrichment analysis revealed strongest enrichment in functional categories related to chromosome organization (*p* = 6.0 × 10^−56^), cell cycle (*p* = 2.0 × 10^−44^), and mitotic cell cycle process (*p* = 2.0 × 10^−42^) in CDC123^high^ TNBC, Fig. 4E, thus corroboration our data.

To identify potentially actionable therapeutic gene targets for BC subtypes, we subsequently interrogated the cancer genes currently listed in the OncoKB precision oncology knowledge base [24], and identified GATA3, CCND1, ESR1, and ELP2 (for HR^+^), ERBB2, and CDK12 (for HER2^+^), and SOX9, HMGA1, ATP1A1, CAD, and PPP1CB (for TNBC) to be known cancer genes, Fig. S4. Among those, GATA3, CCND1, ESR1, ERBB2, and CDK12 are classified as actionable gene targets for potential implementation into patient care. Our data highlight the need for further development and testing of therapeutic agents for the additionally identified genes from the current study for which currently no therapeutic intervention are available.

### Identification of DEGs in BC as function of tumor grade, age, and ethnicity

**Fig. 5:**
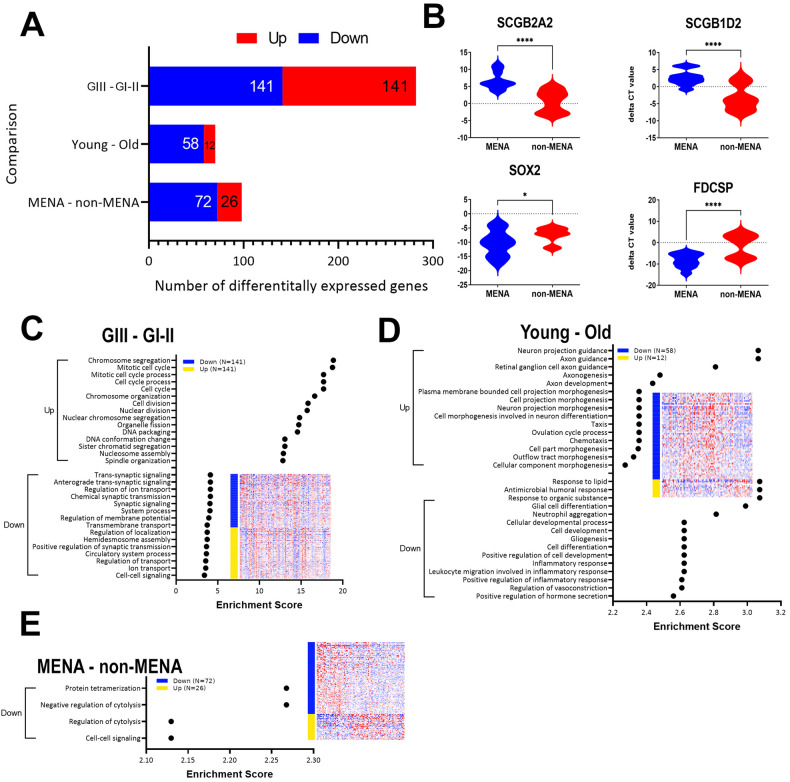
Differential gene expression analysis as function of BC tumor grade, age, and ethnicity. **A** Bar chart depicting the number of DEGs as function of tumor grade (GIII vs GI-II), age (young (≤ 40) vs old (> 40)), and ethnicity (MENA vs non-MENA), using 2.0 fc and adjusted FDR (*p* < 0.05). **B** Validation of two upregulated (upper panel) and two downregulated (lower panel) genes in MENA (*n* = 20) vs non-MENA (*n* = 20) using qRT-PCR where each sample was run in duplicate. Data are presented as violin plot. *p < 0.01, ****p < 0.0001. **C**–**E** Gene ontology (GO) biological processes functional enrichment analysis of upregulated and downregulated genes for each comparison. X-axis depicts enrichment score, while y-axis represents the enriched GO functional category.

Differential expression analysis identified 141 upregulated and 141 downregulated genes as function of tumor grade (GIII vs GI-II); 12 upregulated and 58 downregulated genes as function of patients’ age (young vs old); and 26 upregulated and 72 downregulated genes as function of ethnicity (MENA vs non-MENA), Fig. 5A. The lists of differentially expressed genes as function of tumor grade, age, and ethnicity are provided in Table S6. Upregulated expression of SCGB2A2 and SCGB1D2 and downregulated expression of SOX2 and FDCSP in MENA vs non-MENA was validated using qRT-PCR, which was concordant with the RNA-Seq data (Fig. 5B). Interestingly, when comparing tumors from GIII vs GI–II, we observed an overall enrichment in functional categories associated with mitotic cell division (Fig. 5C). When comparing tumors from young vs old patients, we observed an enrichment of functional categories indicative of neuro projection, axon guidance, ovulation cycle process in young patients, while tumors from old patients were enriched in response to lipid, antimicrobial response, neutrophil aggregation, and other immune-response related functions (Fig. 5D). However, the enrichment significance was modest compared to enrichment as function of tumor grade (Fig. 5C, D). There was minimal enrichment when comparing tumors from MENA vs non-MENA (Fig. 5E). Notably, there was a strong PPI network among downregulated genes in BC from MENA, where inflammation and other functional categories were underrepresented (Fig. S5).

### Differential miRNA expression analysis in BC as function of molecular subtype, tumor grade, age, and ethnicity

**Fig. 6:**
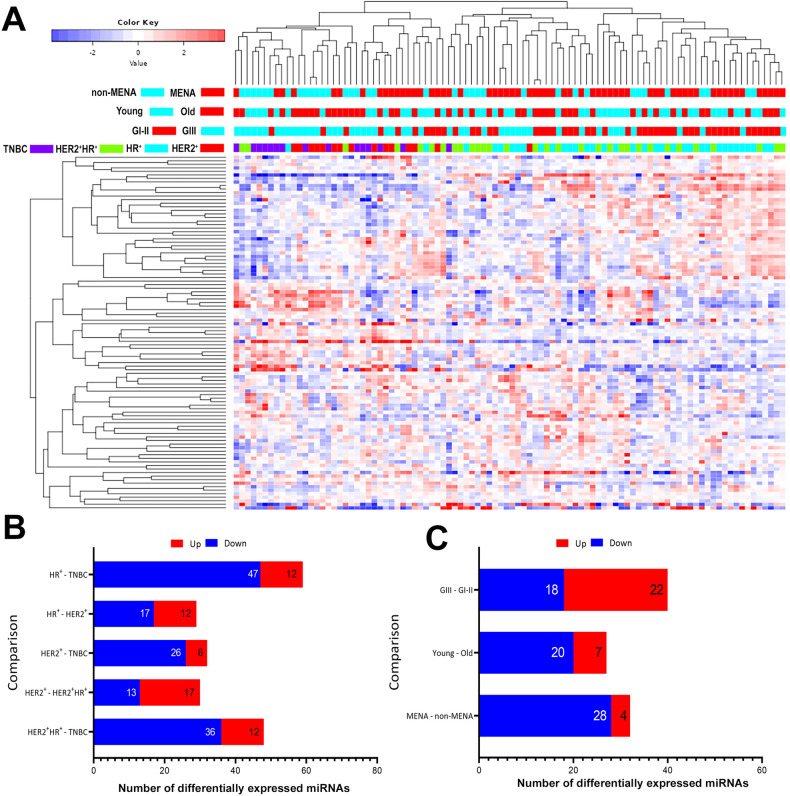
Hierarchical clustering of BC based on miRNA expression. **A** Heatmap depicting clustering of 96 BC patients as function of BC molecular subtype (HR^+^, HER2^+^, HER2^+^HR^+^, and TNBC), tumor grade (GIII vs GI–II), age (young (≤40), old (>40)), and ethnicity (MENA = 60, non-MENA = 36) based on top 100 most variable miRNAs. Color scale depicts the expression level of each miRNA. Each row represents a single miRNA, and each column represents a sample. **B** Bar chart depicting the number of Differential Expressed miRNAs (DEMs) in HR^+^ vs TNBC, HR^+^ vs HER2^+^, HER2^+^ vs TNBC, HER2^+^ vs HER2^+^HR^+^, and HER2^+^HR^+^ vs TNBC^,^ after adjusting for age (young (≤ 40) vs old (> 40)), tumor grade (GIII vs GI–II), and ethnicity (MENA vs non-MENA) using 2.0 fc and adjusted FDR (*p* < 0.05). **C** Bar chart depicting the number of DEMs as function of tumor grade (GIII vs GI–II), age (young (≤40) vs old (>40)), and ethnicity (MENA vs non-MENA), using 1.5 fc and adjusted FDR (*p* < 0.05).

Our previous work has highlighted crucial roles for miRNAs in regulating gene expression in various cancer types, including nasopharyngeal and colorectal [15, 25], as well as their potential use as prognostic marker for BC lymph node metastasis [12]. In the current study, we sought to characterize the potential role of different miRNAs in regulating various aspects of BC. Therefore, the same 96 samples used for total RNA expression profiling were also used for miRNA profiling using the QIAseq miRNA library kit. The data represented in Fig. 6A depicts the clustering of the samples as function of molecular subtype (HR^+^, HER2^+^, HER2^+^HR^+^ and TNBC), tumor grade (GIII and GI–II), age (young (≤40) and old (>40)), and ethnicity (MENA vs non-MENA) based on the top 100 most variable miRNAs. Concordant with the data presented for gene expression, we observed BC molecular subtype to have the greatest influence on clustering, where TNBC and HER2^+^ tumors clustered together, while HR^+^ and HER2^+^HR^+^ tumors clustered separately (Fig. 6A). To some extent, tumors with GIII clustered separately compared to lower grade (GI–II), while age and ethnicity did not seem to have much impact on clustering (Fig. 6A). Using 1.5 fc and adjusted FDR (*p* < 0.05), we identified 12 upregulated and 47 downregulated miRNAs in HR^+^ vs TNBC; 12 upregulated and 17 downregulated in HR^+^ vs HER2^+^, 6 upregulated and 26 downregulated in HER2^+^ vs TNBC, 17 upregulated and 13 downregulated in HER2^+^ vs HER2^+^HR^+^, and 12 upregulated and 36 downregulated in HER2^+^HR^+^ vs TNBC (Fig. 6B). The list of DEMs for each comparison is provided in Table S7. Employing the PAM50 classification, we observed the basal-like and HER2 tumors to cluster separate from LumA, LumB, and normal-like (Fig. S6A) based on miRNA expression, which would be concordant with clustering based on mRNA expression employing the PAM50 classification. Differential expression analysis identified the deregulated miRNAs in each intrinsic subtype, while adjusting for age (young vs old), tumor grade (GIII vs GI–II), and ethnicity (MENA vs non-MENA) using 1.5 fc and adjusted FDR (*p* < 0.05; Fig. S6B and Table S8). Interestingly, differential miRNA expression analysis based on PAM50 classification identified larger number of differentially expressed miRNAs compared to HR and HER2 status classification, which suggest better performance of PAM50 in BC subtype stratification. The largest number of differentially expressed miRNAs was observed when comparing LumA and basal BC, which would be concordant with mRNA differential expression data.

When comparing tumors with GIII vs GI–II, we observed 22 upregulated and 18 downregulated miRNAs, while 7 miRNAs were upregulated and 20 were downregulated when comparing tumors from young vs old (Fig. 6C). Additionally, 4 miRNAs were upregulated, while 28 were downregulated when comparing tumors from MENA vs non-MENA, while adjusting for other confounding variables (Fig. 6C). Overall, the clustering and patterns of DEMs were similar to those seen for DEGs analysis, suggesting essential role for both mRNAs and miRNAs in the pathogenesis of BC.

### Identification of miRNA-mRNA regulatory networks in BC

**Fig. 7:**
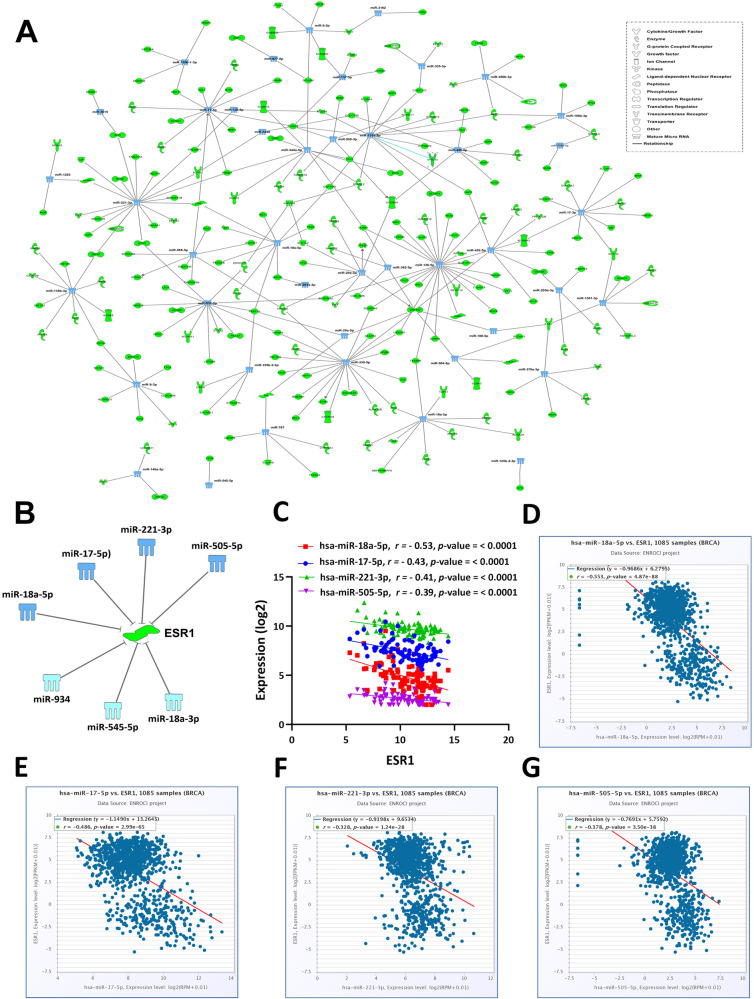
miRNA-mRNA network analysis in HR^+^ BC vs TNBC. **A** Network depicting the interaction between downregulated miRNAs (light blue) and upregulated mRNAs (light green) in HR^+^ vs TNBC based on experimentally validated and highly predicted interactions in IPA. The figure key indicates the class of each miRNA target gene. **B** Illustration of the ESR1-miRNA regulatory network. Light blue color indicates miRNAs with high confidence/experimentally validated, while sky blue color indicates miRNAs with moderate confidence based on IPA analysis. **C** Correlation analysis between the expression of ESR1 and hsa-miR-18a-5p, hsa-miR-17-5p, hsa-miR-221-3p, and hsa-miR-505-5p in the 96 BC samples. **D**–**G** Correlation analysis between the expression of ESR1 and hsa-miR-18a-5p, hsa-miR-17-5p, hsa-miR-221-3p, and hsa-miR-505-5p, respectively in a cohort of 1085 BC patients from TCGA BRCA dataset.

To elucidate the role played by differentially expressed miRNAs in regulating gene expression in different BC molecular subtypes, as function of tumor grade, age at presentation, and ethnicity, we employed the microRNA target filter module in IPA. Our initial analysis investigated the downregulated miRNAs in HR^+^ vs TNBC and the corresponding upregulated genes, in conjunction with predicted miRNA targets (high confidence) and those that have been experimentally validated. Using this approach, we identified 282 miRNA-mRNA interactions (Table S9). When constructed as network, we observed numerous interactions where hsa-miR-135b-5p, hsa-miR-138-5p, hsa-miR-17-5p, hsa-miR-18a-3p, hsa-miR-222-3p, hsa-miR-330-5p, hsa-miR-505-3p, hsa-miR-532-3p, and hsa-miR-9-3p exhibited highest network interactions (Fig. 7A). Interestingly, we observed ESR1, a central player in HR^+^ BC, to be regulated by hsa-miR-17-5p, hsa-miR-18a-5p, hsa-miR-221-3p, and hsa-miR-505-5p (high confidence and experimentally validated), while hsa-miR-934, hsa-miR-545-5p, and hsa-miR-18a-3p were predicted to regulate ESR1 with moderate confidence (Fig. 7B). PPI network analysis on the miRNA-regulated gene targets in HR^+^ vs TNBC revealed central hub involving ESR1, where highest gene set enrichment was for prostate and mammary gland development (Fig. S7). Correlation analysis between ESR1 and hsa-miR-18a-5p, hsa-miR-17-5p, hsa-miR-221-3p, and hsa-miR-505-5p in the 96 BC samples revealed significant (*p* < 0.0001) inverse correlation (Fig. 7C). Concordantly, correlation analysis between the expression of ESR1 and the four miRNAs also revealed significant inverse correlation in the TCGA BC cohort (*n* = 1085), thereby corroborating our findings (Fig. 7D–G).

Similarly, we identified 102 miRNA-mRNA interactions in the upregulated miRNAs and downregulated mRNAs in HR^+^ vs TNBC (Table S10). Network analysis on down- and upregulated miRNAs in HR^+^ vs HER2^+^ and in HER2^+^ vs TNBC and the corresponding up- and downregulated mRNAs revealed multiple miRNA-mRNA interactions as presented in Tables S11–S14. Given the key role for ERBB2 in HER2^+^ BC, hsa-miR-18a-5p was predicted (moderate confidence) to regulate ERBB2 expression. Taken together, our data have highlighted numerous miRNA-mRNA regulatory networks in different BC molecular subtypes.

We subsequently sought to identify the miRNA-mRNA regulatory networks as function of tumor grade. To that end, we crossed the downregulated miRNAs in tumor GIII vs GI–II and their predicted gene targets with the corresponding upregulated mRNAs using IPA. Our data revealed 12 miRNAs (hsa-miR-1-3p, hsa-miR-125b-5p, hsa-let-7a-5p, hsa-let-7c-1-3p, hsa-miR-100-5p, hsa-miR-133a-3p, hsa-miR-153-3p, hsa-miR-204-5p, hsa-miR-30c-5p, hsa-miR-451a, hsa-miR-483-3p, and hsa-miR-485-3p) as key players in regulating the upregulated genes in BC with higher tumor grade (Fig. S8A). Interestingly, hsa-let-7a-5p and hsa-miR-1-3p exhibited the highest interactions. Looking at upregulated miRNAs and the corresponding downregulated mRNAs in GIII vs GI-II tumors, 13 miRNAs (hsa-miR-17-5p, hsa-miR-146a-5p, hsa-miR-3473g, hsa-miR-17-3p, hsa-miR-1260a, hsa-miR-941, hsa-miR-570-3p, hsa-miR-1283, hsa-miR-330-5p, hsa-miR-425-5p, hsa-miR-516a-5p, hsa-miR-18a-5p, and hsa-miR-183-5p) were identified and their interacting bone fide gene targets (Fig. S8B). Taken together, our data have identified a myriad of miRNA-mRNA networks in the context of BC molecular subtype and tumor grade.

### Identification of mRNA and miRNA signatures predictive of RFS

**Fig. 8:**
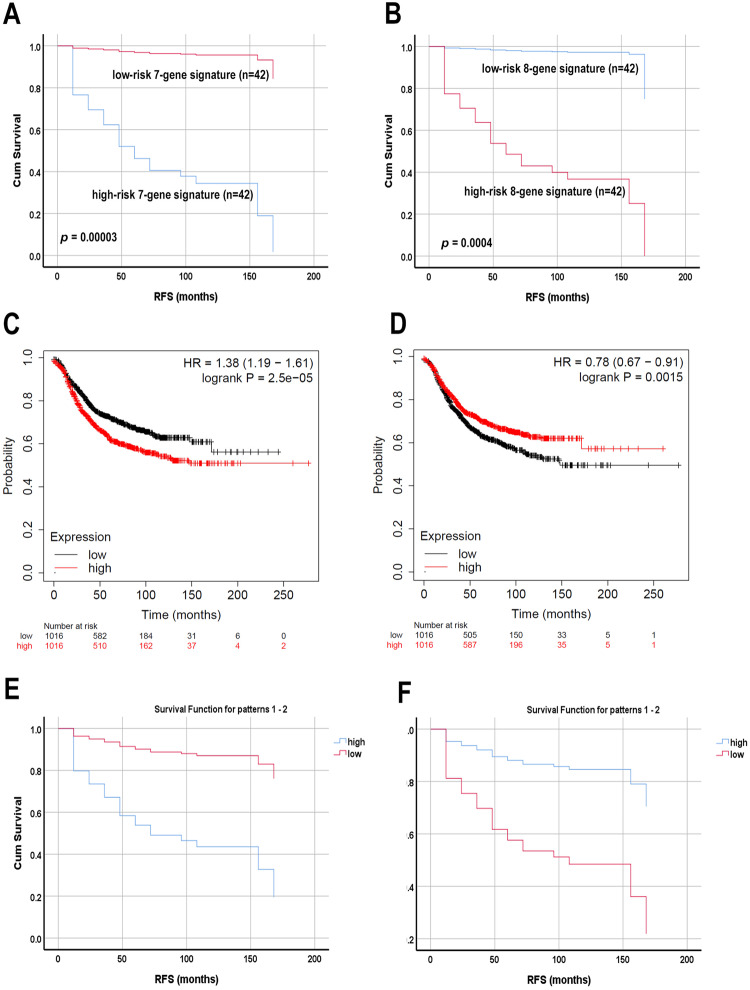
Multivariate Cox regression RFS analysis. Multivariate COX regression RFS analysis for the identified seven-gene **A** and eight-gene signatures **B** predictive of worse and better RFS, respectively, in the same cohort of 84 BC patients. Validation of the worse **C** and better **D** prognostic gene signature in an independent cohort of 2,032 BC patients from the KMplot database. Multivariate COX regression RFS analysis for the four-miRNA **E** and three-miRNA **F** signatures predictive of worse and better RFS, respectively, in a cohort of 84 BC patients.

To identify the set of mRNA transcripts associated with BC patients’ RFS in our cohort, the expression data from a total of 84 patients, for whom long-term survival follow up data is available, were subjected to univariate Cox proportional hazard regression analysis, which identified 555 genes whose expression correlated with patients’ RFS. To adjust for potential confounding variables (tumor grade (GIII and GI-II), molecular subtype (HR^+^, HER2^+^, HER2^+^HR^+^, and TNBC), and age (young and old)), the 555 identified gene were subjected to multivariate Cox proportional hazard model, which identified 176 genes associated with worse prognosis and 218 genes associated with better prognosis (Table S15). In order to identify gene signature predictive of unfavorable and favorable prognosis, the identified 176 and 218 genes associated with variable outcome were subjected to forward stepwise Cox regression model leading to the identification of a seven gene signature (ENSG00000109458(GAB1), ENSG00000109519(GRPEL1), ENSG00000131100(ATP6V1E1), ENSG00000145649(GZMA), ENSG00000177614(PGBD5), ENSG00000187398(LUZP2), and ENSG00000205592(MUC19)) predictive of worse RFS and an eight gene signature (ENSG00000107651(SEC23IP), ENSG00000119950(MXI1), ENSG00000134369(NAV1), ENSG00000141750(STAC2), ENSG00000184117(NIPSNAP1), ENSG00000188352(FOCAD), ENSG00000213967(ZNF726), and ENSG00000254413(CHKB-CPT1B)) predictive of better RFS (Fig. 8A, B). Both signatures were then validated in a large cohort (n = 2032) of BC patients from the KMplot database, which was concordant with our data (Fig. 8C, D). Similar analysis was conducted on expressed miRNAs where multivariate survival analysis identified nine miRNAs (hsa-miR-887-3p, hsa-miR-223-5p, hsa-miR-4677-3p, hsa-miR-3614-3p, hsa-miR-576-3p, hsa-miR-146a-3p, hsa-miR-4772-3p, hsa-miR-7-5p, and hsa-miR-29B-3p) as poor indicators, while eight miRNAs (hsa-miR-130a-3p, hsa-miR-181C-5p, hsa-miR-3117-3p, hsa-miR-181C-3p, hsa-miR-5683, hsa-miR-491-5p, hsa-miR-190b-5p, and hsa-miR-3605-3p) were identified as good prognostic markers in BC, after FDR adjustment. Forward stepwise Cox regression analysis identified a four-miRNA signature (hsa-miR-887-3p, hsa-miR-708-3p, hsa-miR-671-5p, and hsa-miR-146a-3p) predictive of worse RFS and a three-miRNA signature (hsa-miR-5683, hsa-miR-491-5p, and hsa-miR-181C-5p) predictive of better RFS (Fig. 8E, F). Multivariate analysis on the identified mRNA and miRNA signature are provided in Table S16.

## Discussion

BC is a heterogenous disease, where pathological classification of BC according to HR (ER and PR) expressions and HER2 amplification led to significant improvement in the clinical management and tailored treatment choices for patients with BC [26]. In the current study, we employed whole transcriptome analysis, in conjunction with computational and gene set enrichment analysis, to provide a detailed map of mRNA and miRNA expression alterations across BC molecular subtypes, tumors with various disease grade, as function of patients’ age and ethnicity. The most striking difference in gene expression was influenced by BC molecular subtypes, especially when comparing HR^+^ to TNBC and HR^+^ to HER2^+^. Interestingly, we observed HER2^+^ and TNBC to cluster together, while HR^+^ and HER^+^HR^+^ clustered separately. Our data also suggested that the HER2^+^HR^+^ BC resembles HR^+^, rather than HER2^+^ BC, which could have implications on the clinical management of those patients. Our data also identified unique gene signatures associated with each BC molecular subtype. While current treatment choices for each BC molecular subtype has improved the outcome of BC patients [27], there is always a need to develop novel therapeutic targets to improve the efficacy of existing treatment regimens and to overcome lack of response in some patients. Herein, we identified gene sets that are enriched in each BC molecular subtype and additionally identified numerous therapeutic vulnerabilities employing data from the Achilles project and OncoKB. As expected, our data identified ESR1 for HR^+^ and ERBB2, for HER2^+^ tumors, thus corroborating the validity of our approach. Notably, our data also identified numerous additional potential targets for each BC molecular subtype. For instance, we predicted that targeting of LTO1, CCND1, and GATA3 could be more efficient at suppressing HR^+^ tumors than targeting of ESR1 in patients with ER^+^ tumors (Fig. 3E), which remains to be tested in preclinical models. LTO1 is overexpressed in several cancer types and was found to prevent ROS-induced ribosomal damage [28]. CCND1 is a transcriptional target of the ER, which cooperates with CDK4/6 to promote cell cycle progression. Mice lacking CCND1 or CDK4 were resistant to mammary tumor development driven by the HER2 oncogene [29, 30], suggesting CCND1 as potential therapeutic target for HER2^+^ BC as well. While therapeutic targeting of CCND1 is not currently used in the clinic, several small molecular inhibitors targeting CDK4/6 have been developed for the treatment of ER^+^ BC [31]. Numerous studies highlighted a prognostic value for GATA3 in BC, while targeted depletion of GATA3 inhibited BC growth and lung metastasis [32, 33], thus corroborating our findings. Targeting PSMB3, RPL23, RPL19, RPL23A, PSMD3, MED1, and MRPL45 could be more effective than targeting ERBB2 in HER2^+^ BC (Fig. 3F). Interestingly, one study revealed a role for the crosstalk between HER2 and MED1 in promoting resistance to endocrine therapy [34]. While currently TNBC has no targeted therapies, with few exception (i.e., PARP inhibitors for TNBC with defective homologous recombination) [35], our data has identified numerous potential therapeutic targets for TNBC including CDC123, CENPW, PWP2, ATP1A1, NOP2, YBX1, RPP4, PPP1CB, TSPYL5, HMGA1, SLC7A5, ATL2, CAD, CPAMD8, FABP5, SDC1, PPP1R1A, SLC2A1, MRPS17, and SOX9 (Fig. S3). Concordantly, targeted depletion of CDC123 inhibited TNBC CFU potential and inhibited their growth under 3D culture conditions. The potential use of additionally identified novel targets remains to be investigated.

Our data also highlighted numerous miRNA-mRNA regulatory networks in the context of BC molecule subtypes, tumor stage, patients’ age, and ethnicity, thus expanding our knowledge on the role of this class of epigenetic regulators in the pathogenesis of BC. One remarkable finding was the essential role for downregulated miRNAs in regulating potential therapeutic targets in each BC molecular subtype. We observed hsa-miR-17-5p, hsa-miR-18a-5p, hsa-miR-221-3p, and hsa-miR-505-5p, hsa-miR-934, hsa-miR-545-5p, and hsa-miR-18a-3p to regulate ESR1. Additionally, our data revealed regulation of LTO1 by hsa-miR-532-3p and CCND1 by hsa-miR-17-5p in HR^+^ BC. In HER2^+^ BC, our data revealed regulation of RPL23 by hsa-miR-17-3p and hsa-miR-200b-3p, regulation of ALDOC and CACNB1 by hsa-miR-18a-3p, regulation of ACACA by hsa-miR-200b-3p and regulation of ERBB2 by hsa-miR-18a-5p. In TNBC, our data revealed regulation of HMGA1 by hsa-miR-196a-5p and hsa-miR-625-5p, regulation of ATL2 by hsa-miR-31-5p, and regulation of SDC1 by hsa-miR-10a-5p. These data suggest re-expression of those downregulated miRNAs using RNA-based therapeutic as an alternative therapeutic strategy to target HR^+^, HER2^+^, and TNBC BC, respectively.

In addition to the biological and therapeutic insight revealed by our study, we identified seven-gene signature predicative of worse prognosis and an eight-gene signature predictive of better prognosis. Interestingly, the identified gene signatures were validated in a much larger cohort, which suggests genes driving worse and better prognosis are universal, regardless of ethnicity. Similarly, we also identified three and four miRNA signatures predicative of worse and better prognosis, respectively. The prognostic value of the identified miRNAs remains to be validated in additional cohorts.

In the context of ethnicity, our data revealed modest differences between BC from MENA when compared to other ethnicities, when adjusting for BC molecular subtype, grade, and age. Notably, downregulation of SOX2 in BC from the MENA was most profound. This difference is unlikely due to differential expression as function of BC molecular subtype, grade, or age since we have adjusted for those variables during DGE analysis. Nonetheless, we did not observe any correlation between SOX2, ESR1, and ERBB2 expression (data not shown). Numerous studies indicated higher expression of SOX2 to correlate with poor prognosis in multiple cancers, including BC [36]. Those data warrant further validation in larger cohorts and factors leading to suppression of those genes in BC from MENA remains to be identified.

Our data provides a comprehensive map of mRNA and miRNA expression alterations across BC molecular subtypes, tumors with various disease grade, and as function of patients’ age and ethnicity from the MENA region. Our data identified numerous novel therapeutic targets for each BC molecular subtype, which are predicted to outperform existing therapeutic approaches. These findings remain to be tested in preclinical animal models for efficacy and safety. Additionally, our data identified gene and mRNA signatures capable of predicting patients RFS and discriminating each BC molecule subtype, with potential implementation in molecular diagnostics.

## Supplementary information

Reproducibility checklist

Figure S1

Figure S2

Figure S3

Figure S4

Figure S5

Figure S6

Figure S7

Figure S8

Table S1

Table S2

Table S3

Table S4

Table S5

Table S6

Table S7

Table S8

Table S9

Table S10

Table S11

Table S12

Table S13

Table S14

Table S15

Table S16

## Data Availability

All datasets generated or analyzed in this study are provided in this published article and the associated supplementary files. Accession numbers for the RNA-Seq data files generated from current study are provided under materials and methods.
