# Supplementary material for: Transcriptome profiling and network enrichment analyses identify subtype-specific therapeutic gene targets for breast cancer and their microRNA regulatory networks
Source: Cell Death Dis. 2023 Jul 12;14(7):415. doi: 10.1038/s41419-023-05908-8 (PMC10338679; doi:10.1038/s41419-023-05908-8)
Supplement: Supplementary file 2 — Figure S1 [file 41419_2023_5908_MOESM2_ESM.pdf]

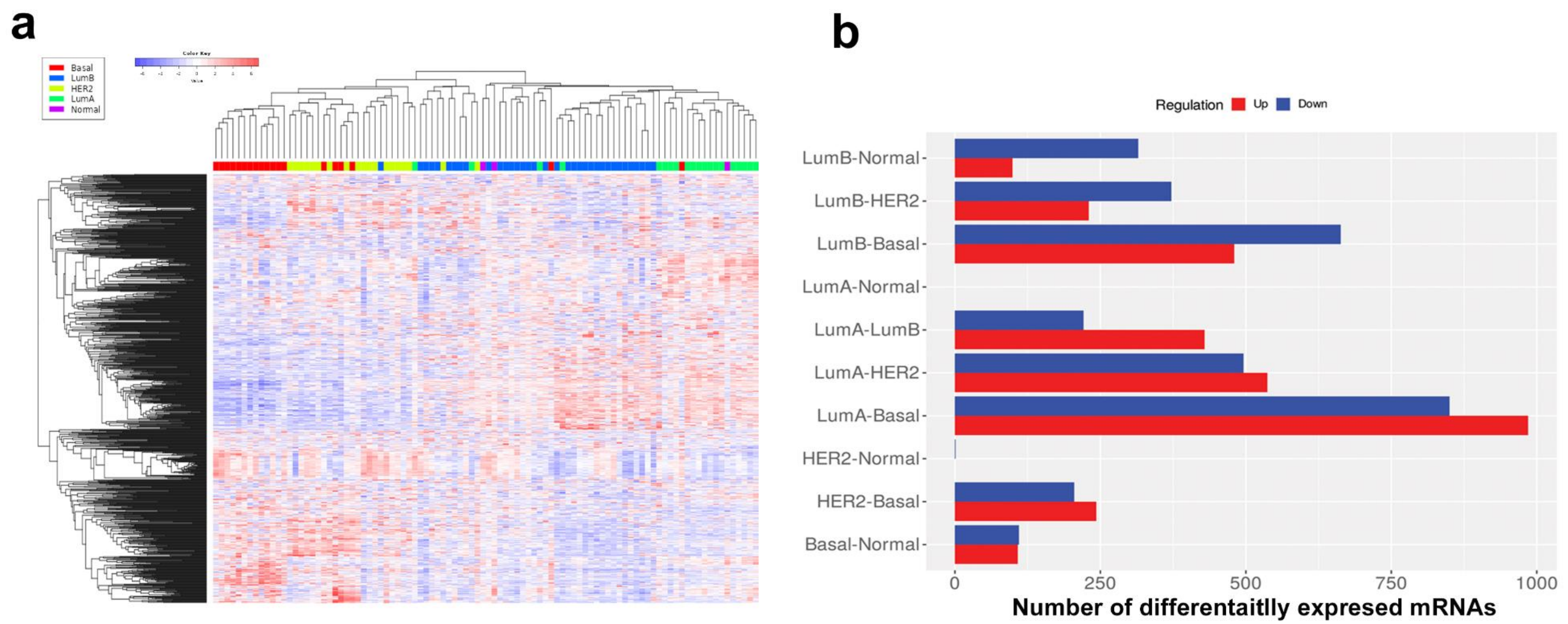

Figure S1. Hierarchical clustering of BC based on mRNA expression as function of PAM50 classification. (a) Heatmap depicting clustering of 96 BC patients as function of molecular subtype (LumA, LumB, HER2, basal-like, and normal-like) based on top 1000 most variable genes. Hierarchical clustering was conducted using correlation distance and average linkage. Color scale depicts the expression level of each gene. Each row represents an mRNA, and each column represents a sample. **(b)** DESeq2 was used to identify differential expressed mRNAs using 2.0 FC and $p < 0.05$ FDR.
