# Supplementary material for: Transcriptome profiling and network enrichment analyses identify subtype-specific therapeutic gene targets for breast cancer and their microRNA regulatory networks
Source: Cell Death Dis. 2023 Jul 12;14(7):415. doi: 10.1038/s41419-023-05908-8 (PMC10338679; doi:10.1038/s41419-023-05908-8)
Supplement: Supplementary file 3 — Figure S2 [file 41419_2023_5908_MOESM3_ESM.pdf]

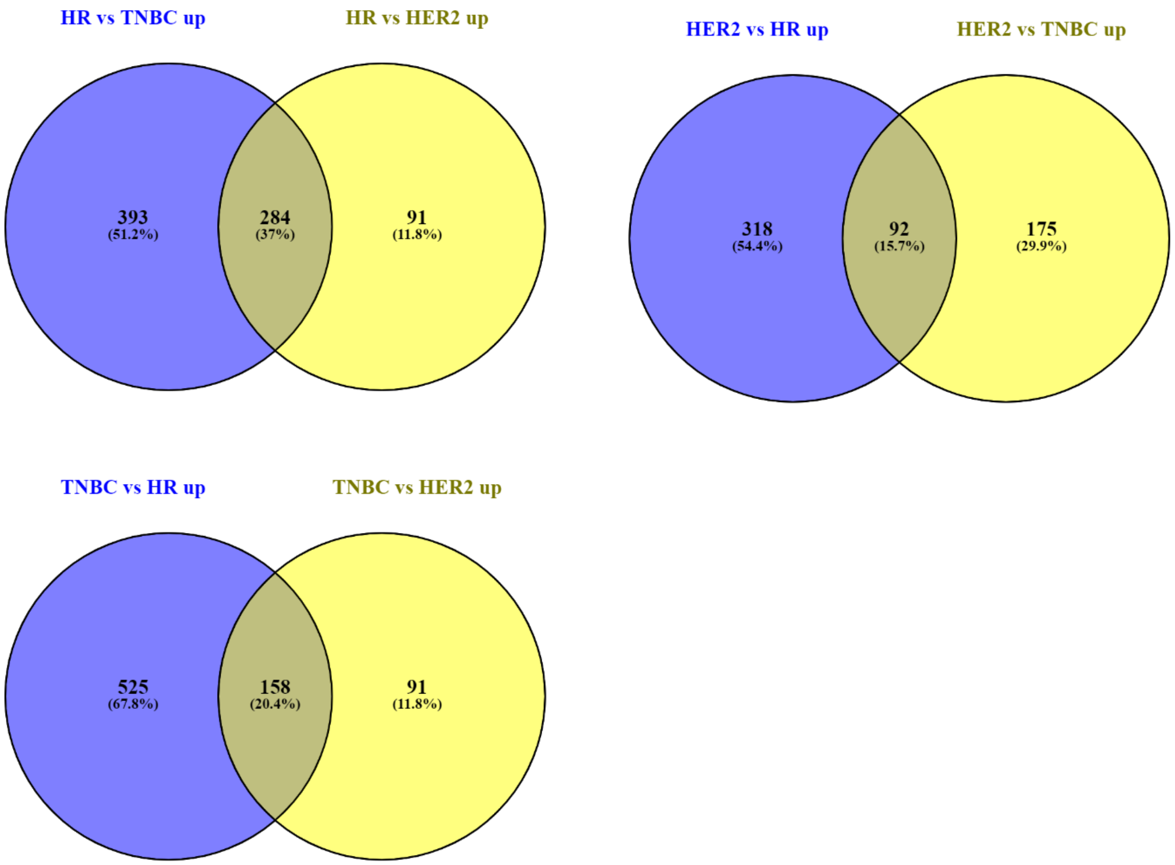

Figure S2. Enriched genes in HR+, HER2+, and TNBC based on DEG analysis. Differential expression analysis identified 284 genes enriched in HR+, 92 genes in HER2+, and 158 genes in TNBC. Those enriched genes were used as input for MarkerFinder prediction.
