# Supplementary material for: Transcriptome profiling and network enrichment analyses identify subtype-specific therapeutic gene targets for breast cancer and their microRNA regulatory networks
Source: Cell Death Dis. 2023 Jul 12;14(7):415. doi: 10.1038/s41419-023-05908-8 (PMC10338679; doi:10.1038/s41419-023-05908-8)
Supplement: Supplementary file 4 — Figure S3 [file 41419_2023_5908_MOESM4_ESM.pdf]

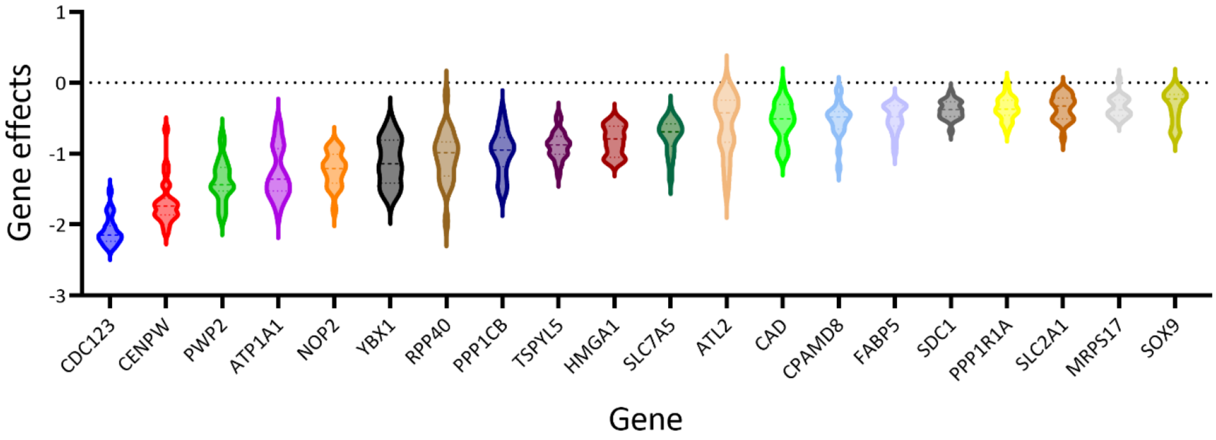

Figure S3. TNBC essential genes. Upregulated genes in TNBC vs HR^+ breast cancer were crossed with TNBC gene effect CRISPR-Cas9 screen data to identify genes essential for TNBC. Y-axis indicated perturbational gene effects.
