# Supplementary material for: Transcriptome profiling and network enrichment analyses identify subtype-specific therapeutic gene targets for breast cancer and their microRNA regulatory networks
Source: Cell Death Dis. 2023 Jul 12;14(7):415. doi: 10.1038/s41419-023-05908-8 (PMC10338679; doi:10.1038/s41419-023-05908-8)
Supplement: Supplementary file 5 — Figure S4 [file 41419_2023_5908_MOESM5_ESM.pdf]

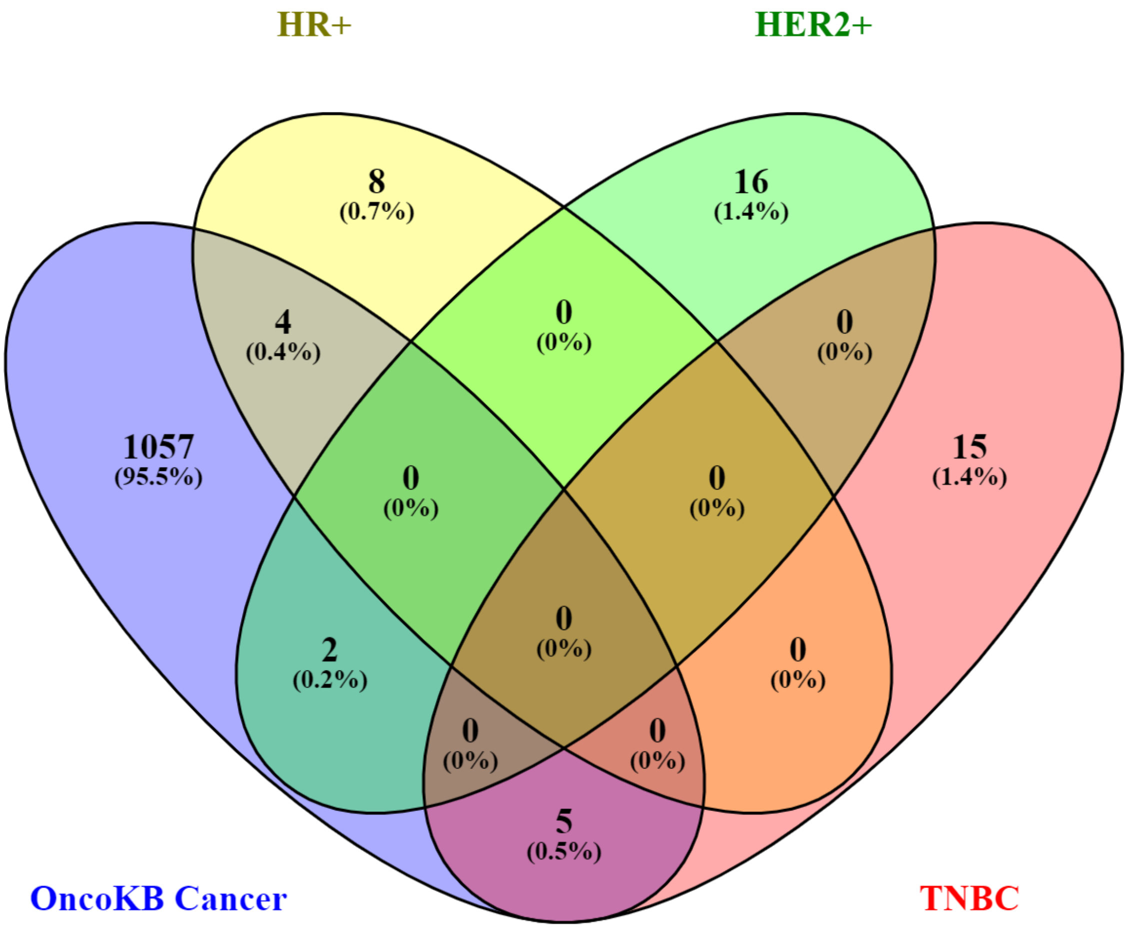

Figure S4. Venn diagram depicting the identified potential gene targets for each breast cancer molecular subtype and cancer genes currently indexed in the OncoKB database.
