# Supplementary material for: Transcriptome profiling and network enrichment analyses identify subtype-specific therapeutic gene targets for breast cancer and their microRNA regulatory networks
Source: Cell Death Dis. 2023 Jul 12;14(7):415. doi: 10.1038/s41419-023-05908-8 (PMC10338679; doi:10.1038/s41419-023-05908-8)
Supplement: Supplementary file 6 — Figure S5 [file 41419_2023_5908_MOESM6_ESM.pdf]

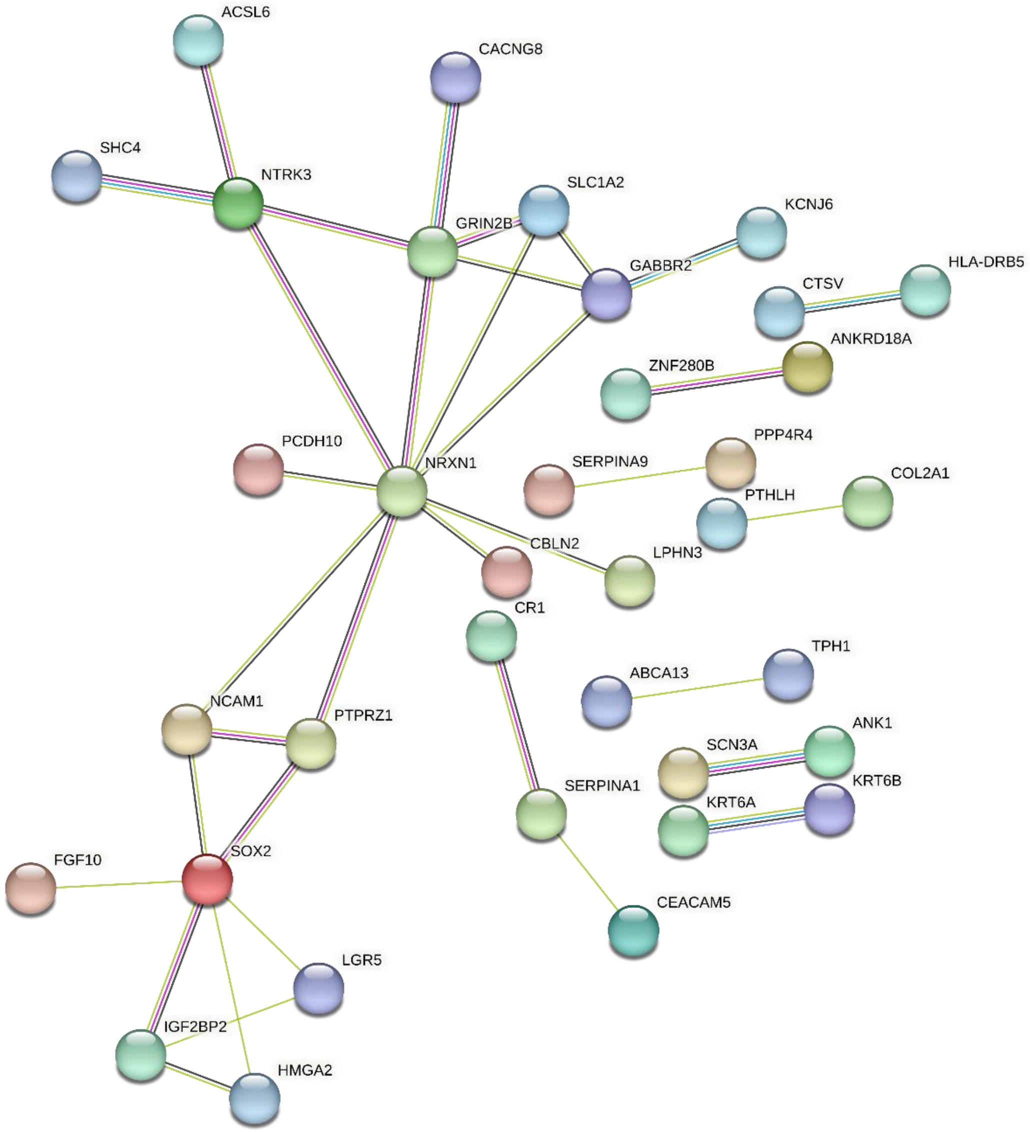

Figure S5. PPI network analysis in downregulated genes in BC from MENA. Seventy-two downregulated genes in MENA (n=60) vs non-MENA (n=36) after adjusting for molecular subtype, tumor stage, and age were subjected to STRING PPI network analysis. PPI enrichment p-value= 1.8×10^{-6}.
