# Supplementary material for: Transcriptome profiling and network enrichment analyses identify subtype-specific therapeutic gene targets for breast cancer and their microRNA regulatory networks
Source: Cell Death Dis. 2023 Jul 12;14(7):415. doi: 10.1038/s41419-023-05908-8 (PMC10338679; doi:10.1038/s41419-023-05908-8)
Supplement: Supplementary file 7 — Figure S6 [file 41419_2023_5908_MOESM7_ESM.pdf]

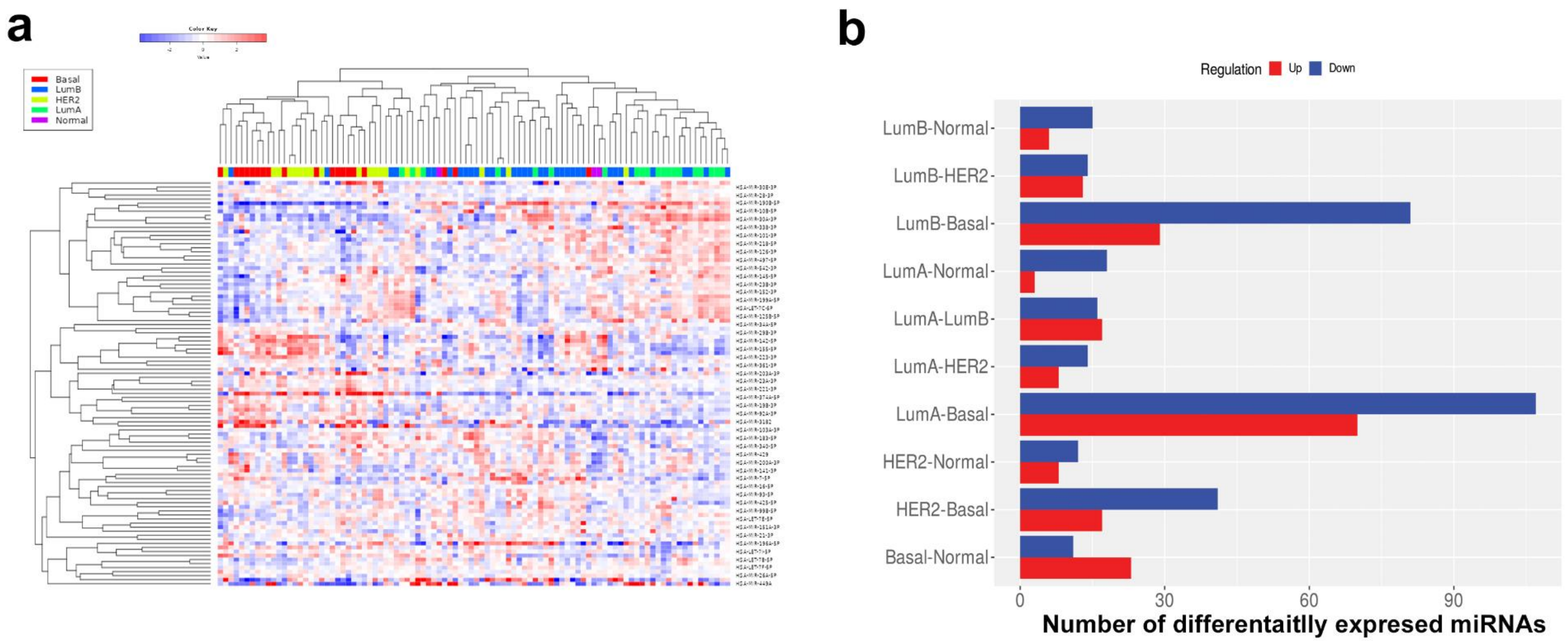

Figure S6. Hierarchical clustering of BC based on miRNA expression as function of PAM50 classification. (a) Heatmap depicting clustering of 96 BC patients as function of molecular subtype (LumA, LumB, HER2, basal-like, and normal-like) based on top 100 most variable miRNAs. Hierarchical clustering was conducted using correlation distance and average linkage. Color scale depicts the expression level of each gene. Each row represents a single miRNA, and each column represents a sample. **(b)** DESeq2 was used to identify differential expressed miRNAs using 1.5 FC and $p < 0.05$ FDR.
