# Supplementary material for: Transcriptome profiling and network enrichment analyses identify subtype-specific therapeutic gene targets for breast cancer and their microRNA regulatory networks
Source: Cell Death Dis. 2023 Jul 12;14(7):415. doi: 10.1038/s41419-023-05908-8 (PMC10338679; doi:10.1038/s41419-023-05908-8)
Supplement: Supplementary file 8 — Figure S7 [file 41419_2023_5908_MOESM8_ESM.pdf]

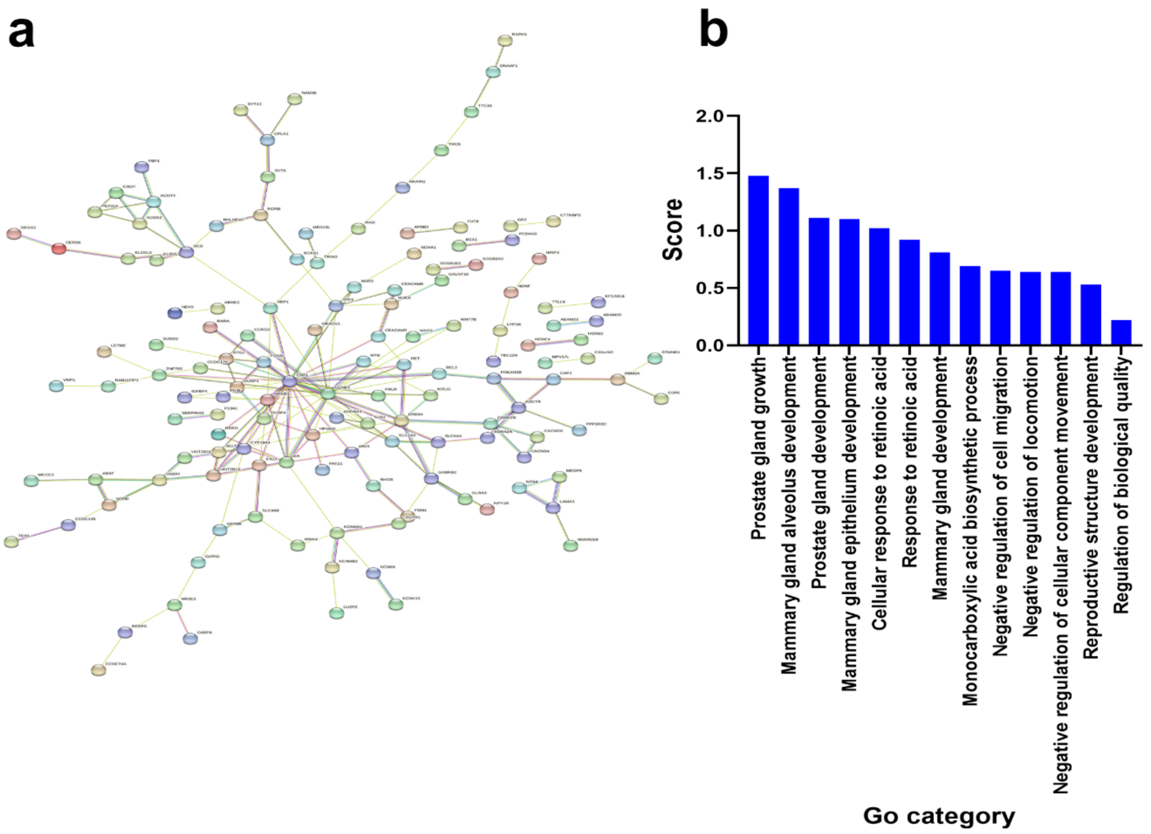

Figure S7. Network analysis of miRNA-targeted genes enriched in HR+ vs TNBC. (a) PPI network analysis of miRNA-targeted genes enriched in HR+ vs TNBC highlighting enrichment in numerous functional categories, including mammary gland development **(b).**
