# Supplementary material for: Transcriptome profiling and network enrichment analyses identify subtype-specific therapeutic gene targets for breast cancer and their microRNA regulatory networks
Source: Cell Death Dis. 2023 Jul 12;14(7):415. doi: 10.1038/s41419-023-05908-8 (PMC10338679; doi:10.1038/s41419-023-05908-8)
Supplement: Supplementary file 9 — Figure S8 [file 41419_2023_5908_MOESM9_ESM.pdf]

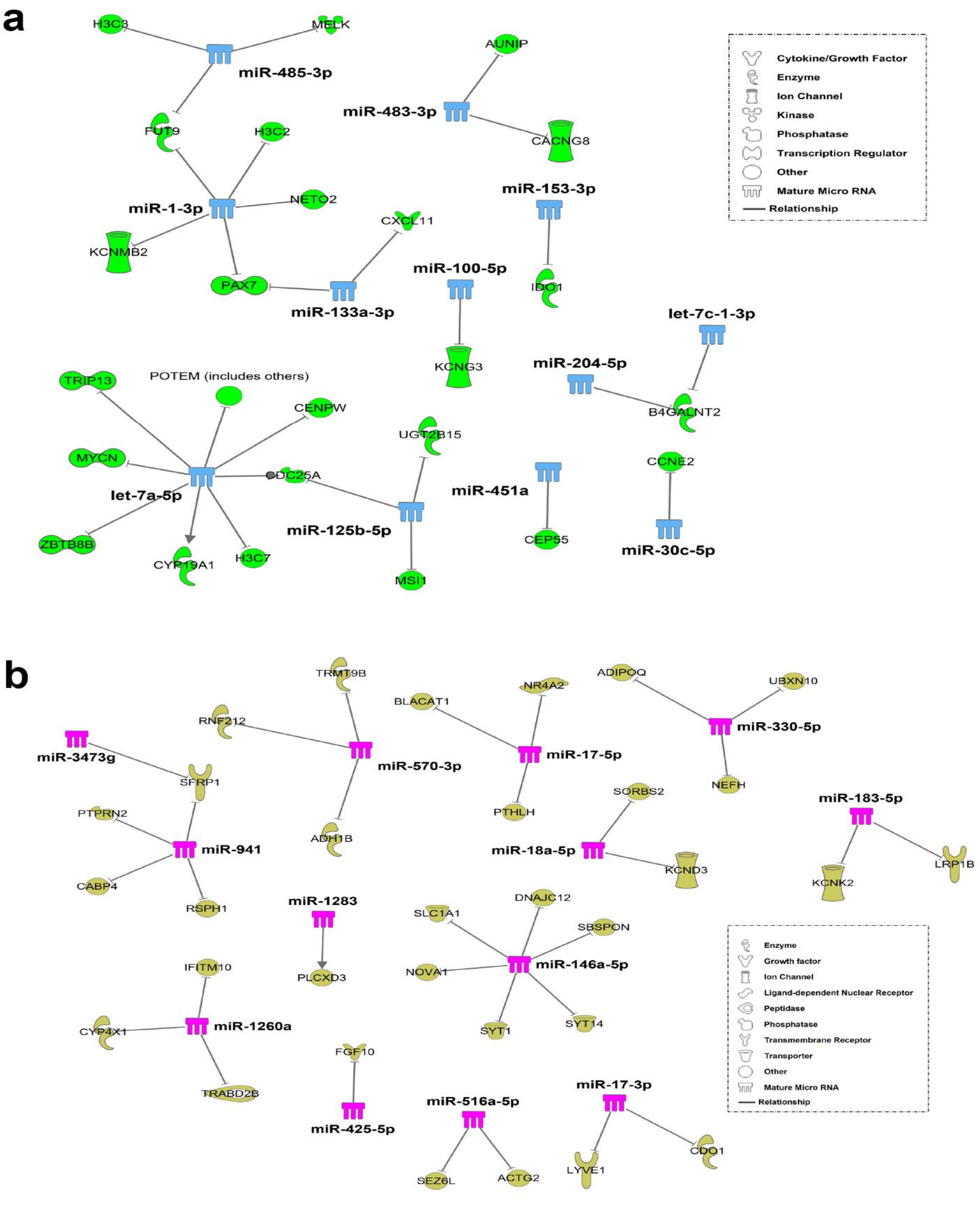

Figure S8. miRNA-mRNA network analysis in breast cancer as function of tumor grade. Network depicting the interaction between downregulated miRNAs (light blue) and upregulated mRNAs (light green) **(a)** or between upregulated miRNAs (blue) and downregulated mRNAs (gold green) **(b)** as function of tumor grade (GIII vs GI-II) based on experimentally validated and highly predicted interactions in IPA. The figure key indicates the type of each miRNA target gene.
