# Supplementary material for: Transcriptome profiling and network enrichment analyses identify subtype-specific therapeutic gene targets for breast cancer and their microRNA regulatory networks
Source: Cell Death Dis. 2023 Jul 12;14(7):415. doi: 10.1038/s41419-023-05908-8 (PMC10338679; doi:10.1038/s41419-023-05908-8)
Supplement: Supplementary file 10 — Table S1 [file 41419_2023_5908_MOESM10_ESM.docx]

| **Table S1.** SYBR green primer sequences used in current study. | | | |
| --- | --- | --- | --- |
| **S.No** | **Names** | **Forward Sequences** | **Reverse Sequences** |
| 1 | GAPDH | 5′-GGAGCGAGATCCCTCCAAAAT-3′ | 5′-GGCTGTTGTCATACTTCTCATGG-3′ |
| 2 | AR | 5′-GACGACCAGATGGCTGTCATT-3′ | 5′-GGGCGAAGTAGAGCATCCT-3′ |
| 3 | FOXA1 | 5′-GCAATACTCGCCTTACGGCT-3′ | 5′-TACACACCTTGGTAGTACGCC-3′ |
| 4 | MYB | 5′-ATCTCCCGAATCGAACAGATGT-3′ | 5′-TGCTTGGCAATAACAGACCAAC-3′ |
| 5 | TFF1 | 5′-GTCCCTCCAGAAGAGGAGTGT-3′ | 5′-GGACTAATCACCGTGCTGGG-3′ |
| 6 | SPDEF | 5′-CAGTGCCCGGTCATTGACA-3′ | 5′-CAGCCGGTATTGGTGCTCT-3′ |
| 7 | SCGB2A2 | 5′-TGGCTGCCCCTTATTGGAGA-3′ | 5′-TGGCATTGTCGTCTATGAACTCT-3′ |
| 8 | SCGB1D2 | 5′-CATTGCGGAAGTCCTGGTGAA-3′ | 5′-AGACAGTGGAAACCAGGATGAA-3′ |
| 9 | SOX2 | 5′-TGGACAGTTACGCGCACAT-3′ | 5′-CGAGTAGGACATGCTGTAGGT-3′ |
| 10 | FDCSP | 5′-GCTGTTGGTTTCCCAGTCTCT-3′ | 5′-ATTGGTGGAAGTGGGCGAAA-3′ |
| 11 | CDC123 | 5′-TCCGAGGCGTTACCATCAAGA-3′ | 5′-ACCAGAGTTCCATCATCGAGTA-3′ |
